# A streamlined workflow for single-cells genome-wide copy-number profiling by low-pass sequencing of LM-PCR whole-genome amplification products

**DOI:** 10.1371/journal.pone.0193689

**Published:** 2018-03-01

**Authors:** Alberto Ferrarini, Claudio Forcato, Genny Buson, Paola Tononi, Valentina del Monaco, Mario Terracciano, Chiara Bolognesi, Francesca Fontana, Gianni Medoro, Rui Neves, Birte Möhlendick, Karim Rihawi, Andrea Ardizzoni, Semini Sumanasuriya, Penny Flohr, Maryou Lambros, Johann de Bono, Nikolas H. Stoecklein, Nicolò Manaresi

**Affiliations:** 1 Menarini Silicon Biosystems spa, Bologna, Italy; 2 Department of General, Visceral and Pediatric Surgery, Medical Faculty, University Hospital of the Heinrich- Heine-University Düsseldorf, Düsseldorf, Germany; 3 Unità Operativa di Oncologia Medica, Policlinico Sant’Orsola – Malpighi, Bologna, Italy; 4 The Institute of Cancer Research and Royal Marsden NHS Foundation Trust, London, United Kingdom; University of Navarra, SPAIN

## Abstract

Chromosomal instability and associated chromosomal aberrations are hallmarks of cancer and play a critical role in disease progression and development of resistance to drugs. Single-cell genome analysis has gained interest in latest years as a source of biomarkers for targeted-therapy selection and drug resistance, and several methods have been developed to amplify the genomic DNA and to produce libraries suitable for Whole Genome Sequencing (WGS). However, most protocols require several enzymatic and cleanup steps, thus increasing the complexity and length of protocols, while robustness and speed are key factors for clinical applications. To tackle this issue, we developed a single-tube, single-step, streamlined protocol, exploiting ligation mediated PCR (LM-PCR) Whole Genome Amplification (WGA) method, for low-pass genome sequencing with the Ion Torrent^™^ platform and copy number alterations (CNAs) calling from single cells. The method was evaluated on single cells isolated from 6 aberrant cell lines of the NCI-H series. In addition, to demonstrate the feasibility of the workflow on clinical samples, we analyzed single circulating tumor cells (CTCs) and white blood cells (WBCs) isolated from the blood of patients affected by prostate cancer or lung adenocarcinoma. The results obtained show that the developed workflow generates data accurately representing whole genome absolute copy number profiles of single cell and allows alterations calling at resolutions down to 100 Kbp with as few as 200,000 reads. The presented data demonstrate the feasibility of the *Ampli*1^™^ WGA-based low-pass workflow for detection of CNAs in single tumor cells which would be of particular interest for genome-driven targeted therapy selection and for monitoring of disease progression.

## Introduction

Chromosomal instability (CIN) and associated chromosomal alterations at focal, arm or entire chromosome level are hallmarks of cancer and play a critical role in solid tumor formation and progression [1–5]. At molecular level, chromosomal alterations modify the genome structure and functions by altering gene transcription, i.e. creating gene fusions between different genes and promoters [6–10], or by altering gene dosage, i.e. through amplifications [11]. Conversely, deletions are important for the inactivation of tumor suppressor genes, such as PTEN and CDKN2A [12,13], and for the elimination of the remaining normal alleles in carriers of inherited or somatic mutations involving RB1, BRCA1, BRCA2, PTPRJ and TP53 [14–17].

A variety of analytical techniques has been developed to analyze chromosomal alterations, such as fluorescence *in situ* hybridization (FISH), metaphase comparative genome hybridization (mCGH) and array-CGH (aCGH). In particular, genome-wide analysis of copy number alterations by aCGH has been widely used to define the copy number landscapes of tumors and has emerged as a powerful tool to identify oncogenes and tumor suppressors, target of recurrent CNAs in tumors, and to study functional relationships in altered genes [18–21]. Analysis of copy number profiles in single tumor cells, supported by the advancements of Next Generation Sequencing (NGS) and WGA technologies in recent years [22–27], has provided insight into different biological aspects of tumor evolution and development. Single tumor cell genome-wide copy number profiling has been used to dissect cancer heterogeneity [28,29], which arises from the reiterative process of clonal expansion, genomic diversification and clonal selection through which cancer evolves [30], and to get a better understanding of tumor evolution [28]. Moreover, molecular characterization of single CTCs released from primary tumors or metastatic sites into the systemic blood circulation has also recently got interest as a biomarker and prognostic factor of response to therapies [31].

However, current methods to generate whole genome libraries from single cells involve several steps from sonication of amplified DNA to fragments polishing and enzymatic adapters ligation [29,32], and are thus not well suited for clinical applications where reproducibility, robustness and rapidity are required. Recently, an optimized library preparation protocol based on a variation of degenerate oligonucleotide primed PCR (DOP-PCR) for highly multiplexed sequencing has been proposed by Baslan et al. However this protocol still requires several enzymatic steps, including WGA adapters digestion, ligation of Illumina^®^-compatible adapters and PCR amplification [33].

In this study, we describe a streamlined workflow for detecting CNAs by low-pass WGS which exploits the characteristics of *Ampli*1^™^ WGA, based on LM-PCR WGA of fragments obtained by digestion on specific restriction sites, to produce, in a single amplification step, barcoded DNA libraries suitable for NGS sequencing. We show that the developed method allows one to obtain an unbiased representation of the original DNA template providing a powerful alternative to widely used aCGH to detect CNAs with high accuracy. Finally, we demonstrate the feasibility of the method proposed for the characterization of CTCs heterogeneity in clinical samples from patients suffering from lung adenocarcinoma or prostate cancer.

## Materials and methods

### Ethic statement

Written informed consent for CTC isolation and genomic characterization was obtained for all patients included. All experiments conformed to the principles set out in the WMA Declaration of Helsinki and were approved by the Ethical Committee Boards responsible for the corresponding studies (Azienda Ospedaliero Universitaria di Bologna, Policlinico S. Orsola Malpighi, Caratterizzazione Molecolare per la Medicina Personalizzata nel Paziente Oncologico, CAMMPPO, 82/2015/O/Tess; Royal Marsden Research Ethics Committee approved protocol CCR2472).

### Cell culture

Cell lines (NCI-H1650, ATCC^®^ CRL-5883^™^; NCI-H1563, ATCC^®^ CRL-5875^™^; NCI-H2228, ATCC^®^ CRL-5935^™^; NCI-H23, ATCC^®^ CRL-5800^™^; NCI-H441, ATCC^®^ HTB-174^™^; NCI-H661, ATCC^®^ HTB-183^™^) were cultured in RPMI 1640 (ATCC modification) supplemented with 10% fetal calf serum (FCS, both Gibco^®^ /Life Technologies^™^) and Penicilline/Streptomicine (Pen/Strep 100x, Euroclone). Cells were maintained at 37°C and 5% CO_2_.

### Cell lines single-cell isolation by micromanipulation

For single cell isolation of mononuclear cells, the bottom of a petri dish was coated with FCS, cell suspensions (cell lines NCI-H1650, NCI-H1563, NCI-H2228, NCI-H23, NCI-H441, NCI-H661) were diluted with 1X PBS to achieve a density of one cell per visual field under an inverse microscope with 10X magnification. Single cells were picked under visual control using a 1 μl pipette and transferred (with 1μl PBS) into a 0.2 ml PCR tube for subsequent whole genome amplification.

### Isolation of patients’ pure CTCs and WBCs by DEPArray

Blood was collected from 1 prostate cancer and 2 lung adenocarcinoma patients. CTCs were enriched (CellSearch CTC Kit, CellTrack^®^ Autoprep, Menarini Silicon Biosystems Inc) and counted (CellTrack^®^ Analyzer II, Menarini Silicon Biosystems Inc) prior to being extracted from CellSearch cassettes and loaded on DEPArray^™^ cartridge (Menarini Silicon Biosystems, SpA, Italy) [34]. With the DEPArray^™^ system, CTCs and White Blood Cells (WBCs) were identified and isolated as pure cells according to the manufacturer’s protocol.

### *Ampli*1^™^ whole genome amplification, DNA library construction and whole genome sequencing

DNA of isolated cells was amplified using the *Ampli*1^™^ WGA kit (Menarini Silicon Biosystems) according to manufacturer instructions. Quality of *Ampli*1^™^ WGA products was checked using *Ampli*1^™^ QC kit (Menarini Silicon Biosystems) and only products with at least 2 amplified bands were retained. 5 μl of *Ampli*1^™^ WGA product were transferred into a new tube and cleaned up with 1.8X SPRIselect Beads (Beckman Coulter) according to manufacturer instructions and eluted in 12.5 μl TE. We designed a streamlined method (which we implemented as a kit called *Ampli*1^™^ LowPass, commercially available from Menarini Silicon Biosystems) for preparing libraries for low-pass WGS by specifically exploiting the deterministic nature of *Ampli*1^™^ WGA. In brief, starting from a 10–50 ng of purified primary *Ampli*1^™^ WGA product, we perform a re-amplification using hybrid PCR primers, including barcoded adaptors compatible with the Ion Torrent^™^ Systems on the 5’ end, and primary WGA universal adaptor on the 3’ end. Barcoded libraries were quantified using Qubit dsDNA HS Assay kit and Qubit 2.0 Fluorometer (Thermo Fisher Scientific) and pooled in equimolar concentrations to obtain 1500 ng in 44 μl of total volume. Pooled libraries were size selected (300–450 bp) using E-Gel SizeSelect^™^ Agarose Gels, 2% on a E-Gel Agarose Gel Electrophoresis System (Thermo Fisher Scientific) according to manufacturer instructions. Size selected library pool was cleaned up with 1.2X SPRIselect Beads (Beckman Coulter) according to manufacturer instructions and quantified using Agilent High Sensitivity DNA Kit using the Agilent Bioanalyzer 2100 instrument (Agilent). Then, library pool was used for emulsion PCR amplification (400bp) and template-positive Ion Sphere Particles (ISPs) were enriched using the Ion Chef^™^ System (Thermo Fisher Scientific). Sequencing was performed using 318 BC chips on the Ion PGM^™^ and Ion 530^™^ chip on Ion S5^™^ System (525 flows).

Libraries from gDNAs (100ng) were prepared using Ion Xpress^™^ Plus gDNA Fragment Library preparation kit (Thermo Fisher Scientific). Briefly, samples were fragmented for 200-base-read libraries, end repaired, ligated with adaptors, nick repaired and bead purified prior to amplification of size selected (E-Gel SizeSelect^™^, Thermo Fisher Scientific) fragments around 250 bp long. Fragment sizes were assessed using the Bioanalyzer system and quantified using the Ion Library TaqMan^®^ Quantitation Kit (Thermo Fisher Scientific). Pooled libraries were used for emulsion PCR amplification (200bp) using the Ion Chef^™^ System (Thermo Fisher Scientific). Sequencing reactions were run on the Ion Proton^™^ System using Ion PI^™^ version 3 chips (Thermo Fisher Scientific).

### Sequence alignment, read counting and normalization

Signal processing, base calling and alignment to *Homo sapiens* hg19 reference sequence was performed with the Torrent Suite^™^ v4.6 with—g 0 parameter for the alignment step with tmap. Genome binning was performed using WindowMaker tool from BEDTOOLS suite [35]. Read counting and assignment to genomic bins were performed using the HTSeq library [36]. Reads spanning more than one bin were assigned to the one with the longest overlap. Read counting and assignment to MseI fragments were performed by BEDTOOLS IntersectBed tool, filtering out reads with more than one fragment match. GC-based normalization was performed by LOWESS fitting of per-bin GC content versus read count on each bin. Calculation of bin mappability value was performed using bigWigAverageOverBed (http://hgdownload.cse.ucsc.edu/admin/exe/) using mappability track for 100mers produced by Encode/CRG (wgEncodeCrgMapabilityAlign100mer; downloaded from https://genome.ucsc.edu/).

### Identification of problematic genome regions

For determination of problematic genome regions, read counts from 21 control WBCs over 500 Kbp bins were GC-normalized and mappability-normalized and divided by median normalized read count. For each bin, the median of normalized read counts across the 21 control WBCs was calculated and bins with median values > 1.4 or < 0.6 were flagged as problematic regions, potentially leading to false positive calls.

### CNA calling

Control-FREEC (Control-Free Copy number caller) software was used to obtain copy-number calls, using the mode without control sample [37]. Read counts were corrected by GC content and mappability (uniqMatch option). Bin size was manually set in order to match the desired resolution. To determine significant CNA calls, Wilcoxon test and Kolmogorov-Smirnov test (p value < 0.01) were performed using the script assess_significance.R provided with Control-FREEC software.

### ROC curves

To assess the sensitivity and specificity of single cell low-pass experiments, the altered copy number status on each single cell was compared, in windows of 500Kbp, to the CNA calls of their corresponding reference WGS of non-amplified gDNA of the respective cell line by means of a receiver operating characteristic (ROC) curve. The comparison refers only to the presence of a CNA in the single cell data versus the reference. Type (gain or loss) and actual copy number were not considered in the comparison. Computation of true and false positive rates for various Wilcoxon non-parametric p-value thresholds and the area under the curve (AUC) were performed using scikit-learn python library. Analogous analyses were performed also to assess sensitivity and specificity at variable read depths, using a 3.5 million reads dataset as reference, and to assess sensitivity and specificity of *Ampli*1^™^ LowPass protocol respect to aCGH.

### Ploidy determination

To determine the ploidy of single cells, raw BAM data were processed as follows:

1. GC-normalized, mappability-normalized and median centered read counts over 500 Kbp bins were multiplied by the ploidy to be tested and were smoothed using the method implemented in smoothseg R package [38] to reduce random noise of NGS data obtained at shallow coverage, which may affect the later determination of ploidy levels [39].
2. A probability density function was estimated from the smoothed data using kernel density estimation (KDE); KDE bandwidth is estimated by Silverman’s ‘rule of thumb’ [40] and, if necessary, manually tweaked after visual inspection of the density plot to best reflect underlying data distribution.
3. Estimates of the copy numbers are obtained by finding peaks on the KDE fitted data as described by Du et al. [41]; peaks with a relative probability contribution lower than 2% are excluded as potential false positives.
4. Copy number estimates were rounded to the nearest integer and resulting values are assumed to be the putative underlying copy numbers. Given the discreet nature of read counts, which are expected to be directly proportional to DNA content, the copy number estimates should increase linearly with the underlying copy number. The estimates were thus fitted to a linear regression *y* = aP* where *a* is the slope for P, which is a vector of the putative copy numbers.
5. Process was repeated for each ploidy to be tested (from 2 to 8)

Only main ploidies for which R^2^ > 0.98 were considered further and best fitting main ploidy was selected based on sum of squared residuals (SSR). Since ploidies multiple of the real main ploidy would produce similar fittings and SSR values, results are manually reviewed and the lowest possible plausible ploidy with similar SSR and R^2^ values was selected.

### Comparative genomic hybridization with oligonucleotide microarrays (aCGH)

aCGH analyses on oligonucleotide arrays were performed according to the manufacturer’s instructions (Agilent Oligonucleotide Array-Based CGH for Genomic DNA Analysis, Version 6.4, August 2011, G4410-90010) with slight modifications as described in [42]. All CGH arrays were processed using the Microarray Scanner G2565CA by Agilent Technologies with 3 μm resolution and 16 bit color depth. The output image files were imported, normalized and fluorescent ratios for each probe were determined using Feature Extraction software (Agilent Technologies, Version 10.7.3.1, Protocol CGH_107_Sep09). Feature Extraction output files were imported into the Genomic Workbench 5.0.14 software. aCGH data were examined using the aberration detection method 2 (ADM-2) algorithm with a threshold of 6.0. No centralization was applied. An aberration filter was defined for identifying copy number alterations, where changes only were considered as true positive events with a minimum log2ratio of 0.3 and a minimum of 50 consecutive probes with the same polarity per region.

## Results

### Development of an *Ampli*1^™^-based protocol for low coverage whole genome sequencing (*Ampli*1^™^ LowPass)

**Fig 1:**
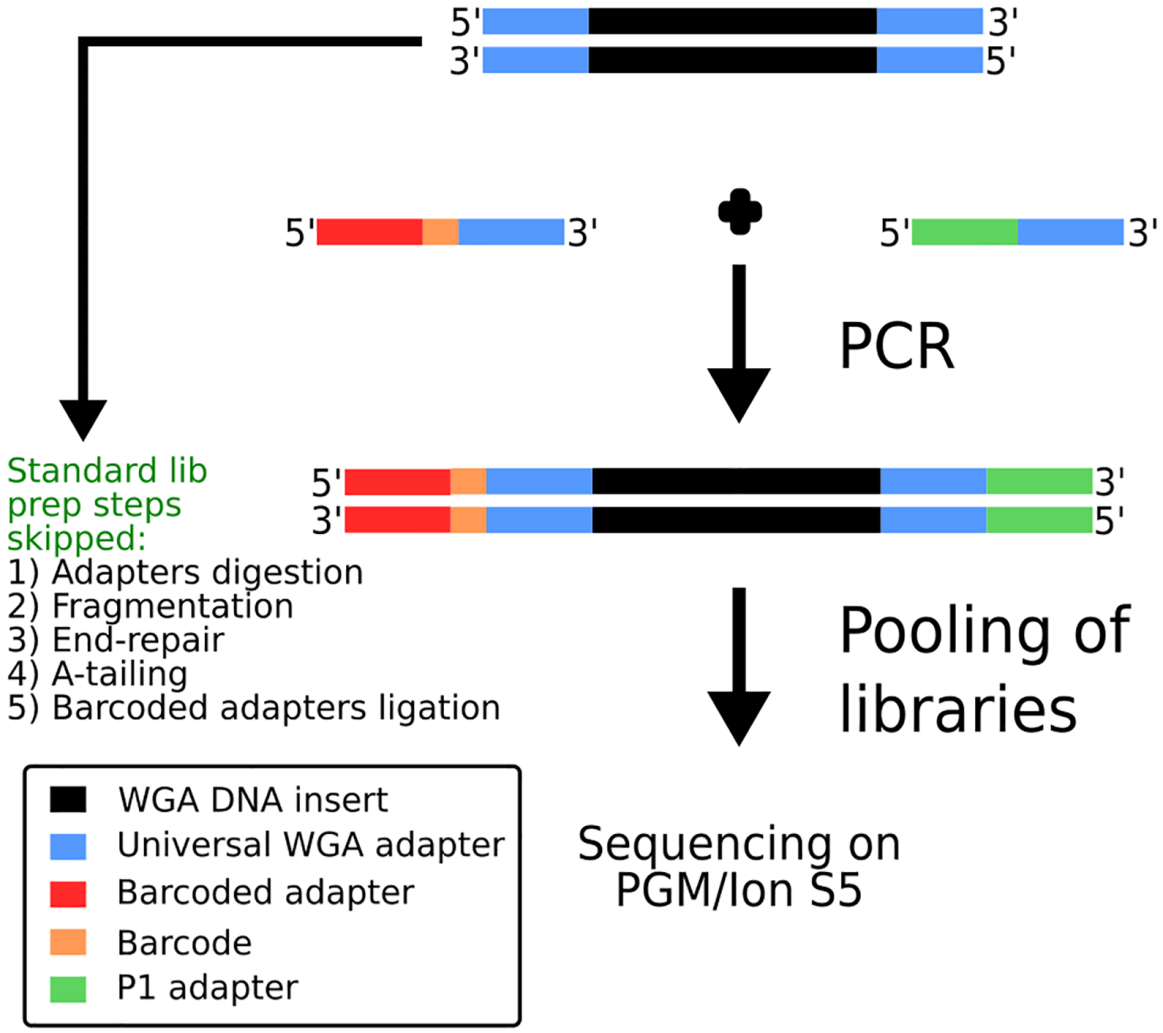
Schematic overview of *Ampli*1^™^ LowPass approach. DNA is amplified through primers complementary to *Ampli*1^™^ WGA universal adapters through a single PCR reaction. Primers incorporate Ion Torrent^™^-compatible adapter sequences and barcodes. Libraries are then pooled and subjected to standard processing for sequencing on PGM or Ion S5 platforms.

*Ampli*1^™^ WGA, based on LM-PCR, was employed for DNA amplification because it showed accurate and more even representation of the original single-cell genomic DNA compared to available methods, as shown by previous reports [27,43,44]. *Ampli*1^™^ WGA has already been used for low-pass WGS by Hodgkinson C. L. et al. [31]. However, according to the workflow used in that paper, the creation of Illumina^®^-compatible libraries requires several steps including i) digestion of WGA adaptors, ii) DNA fragmentation, iii) EndRepair iv) A-Tailing v) barcoded adaptor ligation, vi) sample pooling of barcoded NGS libraries and vii) sequencing. To avoid complex processing steps and streamline the protocol we devised a method, named *Ampli*1^™^ LowPass, which exploits the universal sequences at the end of *Ampli*1^™^ WGA DNA to incorporate Ion Torrent^™^ compatible adapters (Fig 1). A single PCR amplification step is employed to produce barcoded libraries which are ready to be pooled for sequencing, thus skipping laborious and costly processing steps. The same amplification also introduces barcodes incorporated into one of the primer sequences as shown in Fig 1. Finally only a size selection step is needed to make the libraries compatible with the sequencing platform (Ion Torrent^™^ PGM or IonS5).

### *Ampli*1^™^ LowPass produces a comprehensive representation of the human genome

**Fig 2:**
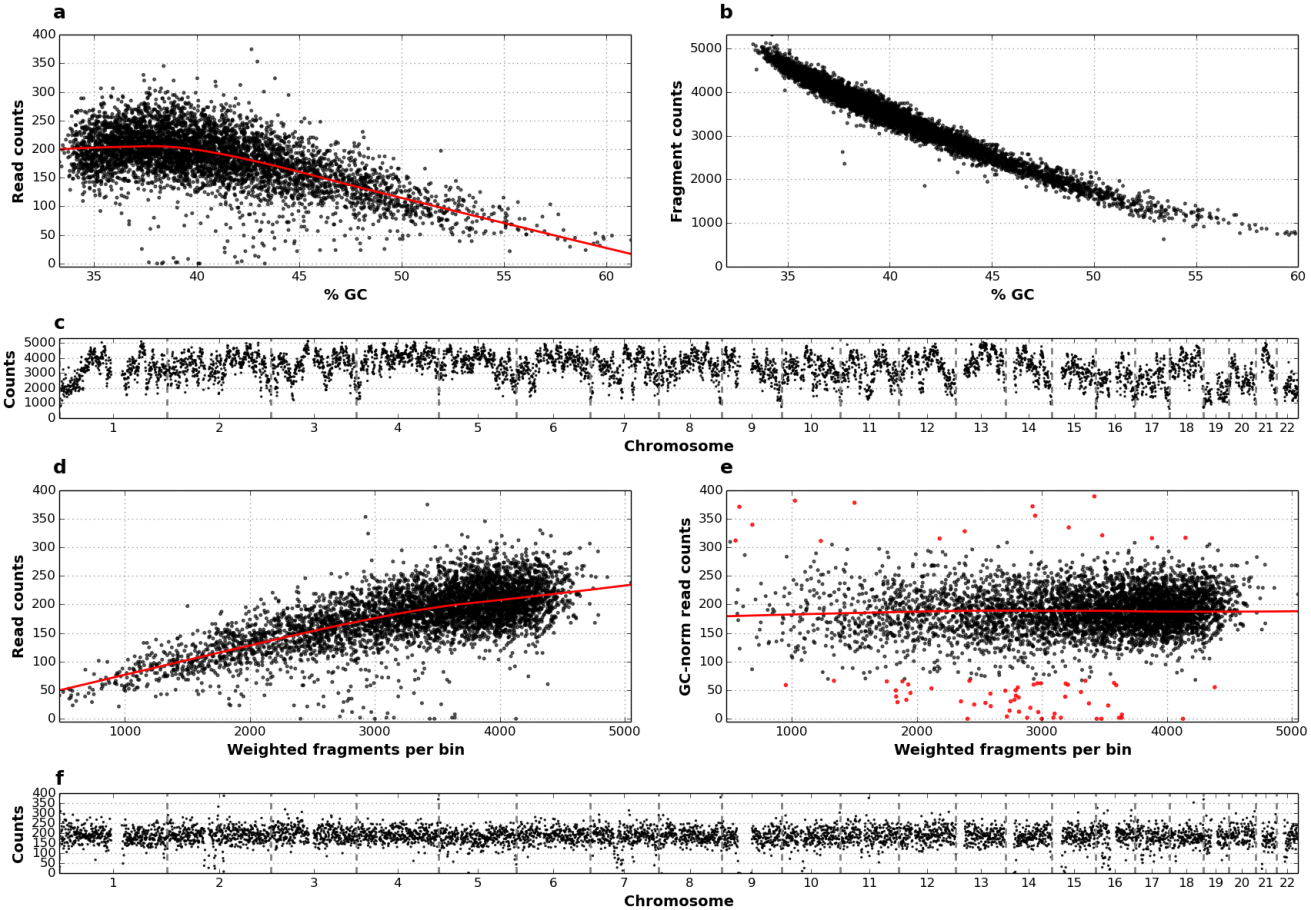
Effect of normalization on read counts distribution. a) Scatter plot of read counts, normalized on 1 million of reads, versus GC content in 500 Kbp bins obtained by sequencing of a single WBC; number of *MseI* fragments per bin is plotted b) respect to GC content and c) along the 22 autosomes; scatter plots of read counts in a single WBC versus number of *MseI* fragments per bin, weighted on per-fragment probabilities, before d) and after e) GC normalization, three standard deviations are used to discriminate outliers (red dots); f) GC-normalized read counts plotted along the 22 autosomes.

To assess the presence of biases and determine the uniformity of genome representation by *Ampli*1^™^ WGA size-selected fragments, genomic DNA from 21 WBCs from 8 individuals was amplified and sequenced on Ion Torrent^™^ PGM platform. On average, 528,836 reads were generated per sample (S1 Table). Genome was divided into 500kb fixed-size, non-overlapping bins. Read counts per bin showed a strong dependency on GC content (Fig 2a). This is however expected as it is well known that read counts are affected by polymerase biases in presence of high or low GC content [45–47]. Another potential source of read count bias is the non-homogeneous distribution of *MseI* sites (TTAA) along the genome, also dependent on GC content (Fig 2b), which leads to different numbers of fragments generated from different genomic regions (Fig 2c). Moreover, fragments generated by *MseI* restriction employed in *Ampli1*^™^ WGA kit are size selected prior to PCR amplification, potentially leading to further bias in read counts. To evaluate this source of bias and effect of GC-based normalization, we calculated the number of fragments per bin, weighted on the fragment length probability (S1 Fig) and evaluated the correlation with read counts before and after GC normalization. As expected, plot of raw read counts against the weighted number of fragments per bin showed a strong bias (Fig 2d). However, biases in read counts were effectively corrected by GC-based normalization (Fig 2e), currently implemented in available software for CNA detection from WGS data [37,39,48], improving the distribution of read counts and producing an even and tight normalized read count distribution along the genome (Fig 2f). Corrected data showed also a high consistency of read count distribution across different control WBCs (S2 Fig).

After normalization, a minor number of bins corresponding to about 1.5–2% of the genome still showed a high deviation (> 3*standard deviation) from the median of normalized counts, likely due to poor mapping in correspondence of repeated and low complexity regions (Fig 2e, red dots). Indeed, the analysis of the distribution of mappability values for highly deviating bins confirmed that they mainly correspond to regions with low mappability values (S3 Fig). Mappability-based normalization is also currently implemented in available software for CNA detection from WGS data [37,48].

To evaluate the homogeneity of genome representation, we calculated a uniformity value defined as the fraction of bins whose normalized read count is at least 20% of the normalized read count averaged across all the bins. On average, uniformity greater than 99% was obtained for each sample (S4 Fig) and, furthermore, more than 90% of bin bases were covered at higher than 60% of the mean coverage in all the control samples, implying a very tight distribution of normalized read counts around the mean.

Finally to identify problematic regions we analyzed the median of the normalized fold change across 21 WBC normal controls. Bins, with normalized read depths highly deviating (FC ≥ 1.4 or FC ≤ 0.6) from the genome median value, were mostly located near pericentromeric and telomeric regions, which are typically rich of repeated and low-complexity regions (S5 Fig). A list of 50 problematic regions with size up to 2 Mbp, and globally accounting for 28 Mbp, was built and was used in following analyses to filter false positive CNA calls.

### *Ampli*1^™^ LowPass produces distortion-free accurate copy number profiles

**Fig 3:**
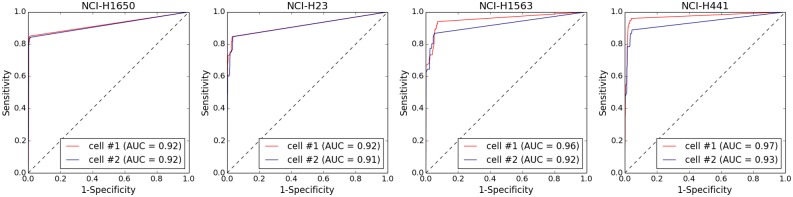
Performance of CNA calling in amplified vs. non-amplified DNA in 4 aberrant cell lines. Data obtained by low-pass WGS (0.5-1M reads) of DNA from single cells amplified with *Ampli*1^™^ WGA kit were processed for CNA calling. CNAs detected in non-amplified bulk gDNA (20-30M reads) were used as reference. For all the 4 cell lines considered ROC analysis showed an excellent agreement (0.91≤AUC≤0.97) between CNA calls from single cells and bulk gDNA.

To verify absence of distortions and residual biases in copy number profiles due to *Ampli*1^™^ WGA of single-cell DNA, we compared the profiles generated by *Ampli*1^™^ LowPass of 2 single cells from each of 4 aberrant cell lines (NCI-H1650, NCI-H1563, NCI-H23, NCI-H441) with those generated by WGS sequencing of the corresponding bulk genomic DNA. WGS of genomic DNA from the 4 cell lines generated between 20,4 to 31.9 million reads, while from 633,049 to 1,284,763 reads were generated from sequencing of the amplified DNA from the single cells (S1 Table). Copy number profiles were generated from GC-normalized and mappability-normalized read counts in 500 Kbp bins. Visual inspection of the profiles showed a strong agreement between copy number profiles generated from single cells and bulk genomic DNA (S6–S9 Figs). To analyze more in depth the agreement between CNA calls in single cells and corresponding bulk DNA we performed a ROC analysis, using Wilcoxon non-parametric test as classifier, to call for copy gains and losses. For all the 4 cell lines analyzed, AUCs ≥ 0.91 were obtained indicating a strong agreement between CNA calls from *Ampli*1^™^ LowPass of single cells and corresponding bulk DNA (Fig 3). Above data confirms WGA does not introduce any significant bias in whole genome copy number profiles and produces accurate CNA calls.

### Optimization of coverage and resolution

To determine the number of reads necessary to reach high specificity and low number of false positives we merged the alignments from the 21 WBCs and randomly subsampled the dataset in subsets ranging from 100,000 mapped reads to 8 million mapped reads. Data analysis was performed at different bin sizes, corresponding to different resolutions, ranging from 100 Kbp to 2 Mbp. Resulting copy number profiles are expected to be free from CNAs. Any call was treated as a false positive call and specificity was calculated. Interestingly, at 200 Kbp resolution, 400,000 reads are sufficient to get specificity = 0.994 (S10 Fig), which shows a dependency on both read number and bin size.

**Fig 4:**
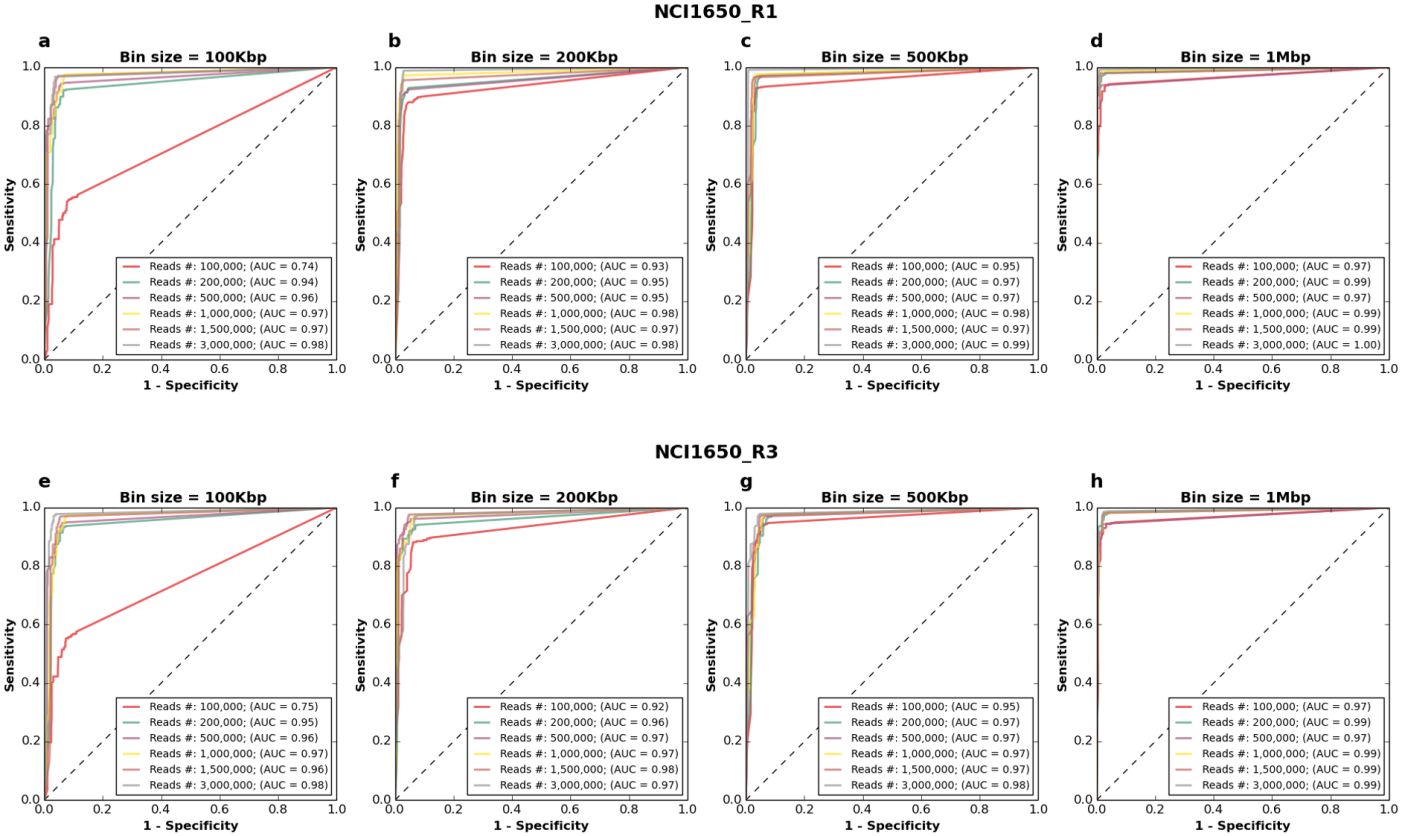
CNA detection by low-pass experiments at different read depths and resolution. Two cells (a-d & e-h) from cell line NCI1650 were analyzed at different window size/resolutions (a,e = 100Kb; b,f = 200Kb; c,g = 500Kb; d,h = 2,000Kb). A dataset at 3,500,000 reads served as reference for ROC analysis.

To estimate sensitivity, specificity and accuracy of CNA calling depending on read number and resolution, DNA from two single cells from the aberrant cell line NCI-H1650, deriving from metastatic site in state 3B adenocarcinoma, was processed with *Ampli*1^™^ LowPass protocol (S1 Table). Following mapping to hg19 reference genome, subsets ranging from 100,000 to 3.5 million mapped reads were extracted by random sampling of alignments and CNAs were called at different resolutions ranging from 100 Kb to 2 Mbp (S11–S18 Figs). It is to note that, while longer bins may miss smaller CNAs, on average 93.88% and 92.74% of the total length of CNAs detected at a resolution of 100 Kb were also detected at resolutions of 500 Kbp and 1 Mbp respectively, using a dataset of 3.500.000 mapped reads. This is expected as most CNAs in tumors are in the order of megabases [18]. A ROC curve analysis was performed using the dataset at 3.5 million mapped reads as a reference. For both cells, 200,000 mapped reads were sufficient to get an excellent accuracy at all the resolutions tested with AUCs ranging from 0.94 to 0.99 (Fig 4) as confirmed also by visual analysis of copy number profiles (S11–S18 Figs). For subsamples of 100.000 reads, we observed a decrease of the AUC by 25% at 100 Kbp bin size, thus indicating that lower bound for accurate CNAs detection at 100.000 reads is approximately 200 Kbp. From a quantitative point of view, copy number changes at a resolution of 500 Kbp were also maintained consistently across the different subsets, showing a mean R^2^ of 0.94 and 0.89 between copy number profiles obtained from 3.5 million mapped reads with those obtained from 1 million and 0.5 million mapped reads respectively (S19 Fig). Correlation, however, decreased rapidly at lower resolutions and read counts likely due to stochastic noise in read counting.

To conclude, at a resolution of 500 Kb, sufficient to resolve the majority of CNAs in tumors, 500,000 mapped reads are enough to get an accurate CNA calling both from a qualitative and quantitative point of view. At lower bin sizes it is still possible to get an accurate detection of aberrations even if profiles start to get noisier from a quantitative point of view for low read coverages.

### Comparison with aCGH

**Fig 5:**
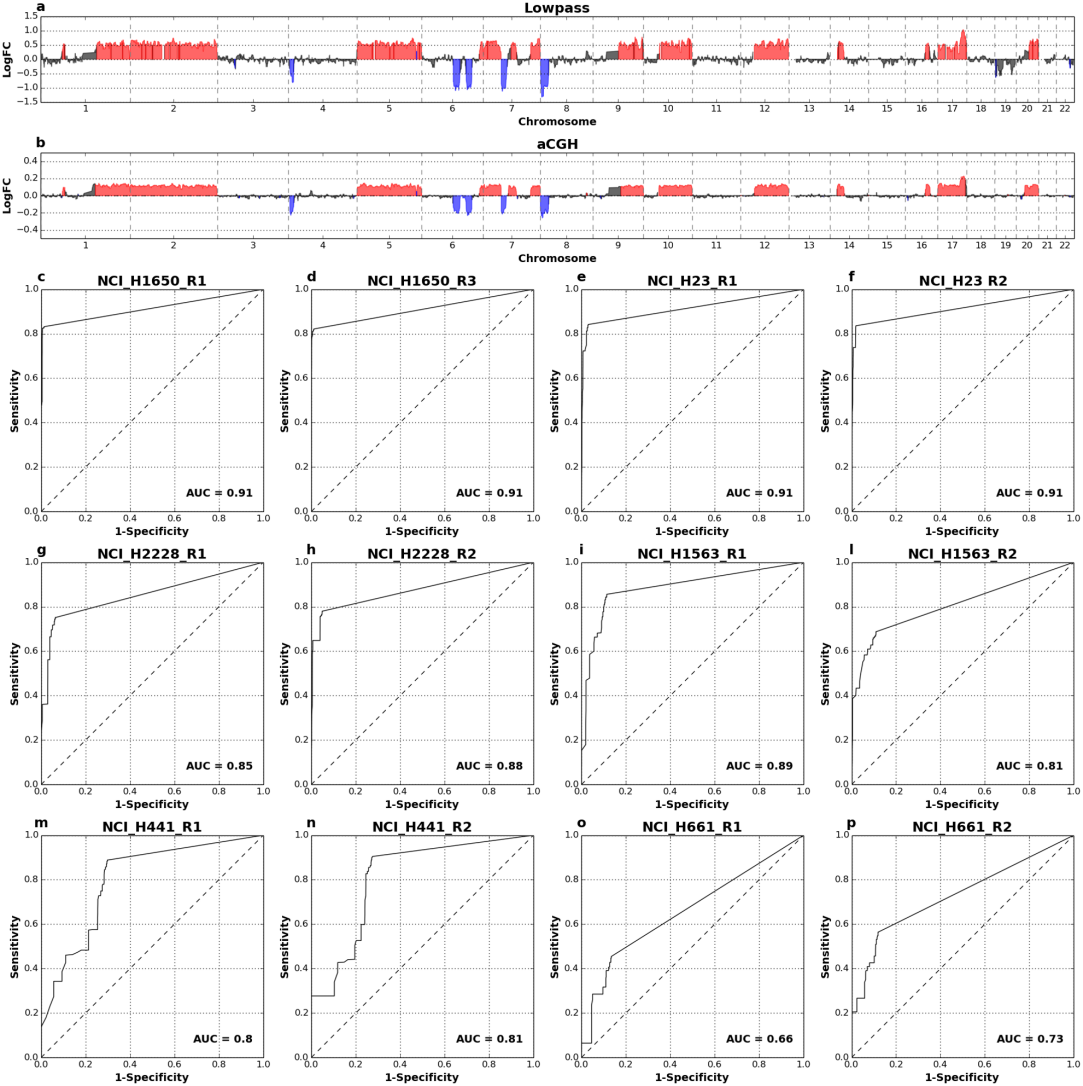
Comparison of LowPass copy number profiles and CNA calling with aCGH. Example profiles from one single cell of aberrant cell line NCI-H23 generated by *Ampli*1^™^ LowPass (a) and aCGH of *Ampli*1^™^ amplified DNA (b). In c-p): ROC curves comparing *Ampli*1^™^ LowPass CNA calls with aCGH calls from single cell of 6 cell lines of the NCI-H series.

aCGH is a widely used and accepted method for screening CNVs and CNAs in clinical diagnostics [49]. Moreover a previous report has shown that, in conjunction with *Ampli1*^™^ single-cell WGA technology, aCGH provides precise and high resolution assessment of copy number changes in single cells [42]. To compare CNA calling by *Ampli*1^™^ LowPass with aCGH, DNA from 6 aberrant cell lines (NCI-H1650, NCI-H23, NCI-H2228, NCI-H1563, NCI-H441, NCI-H661) was amplified with *Ampli1*^™^ WGA kit and processed with both aCGH (G3 4x180k Agilent aCGH microarrays) and low-pass protocols. Based on the above results we aimed at producing about 500.000 reads per cell and we analyzed copy number profiles with a bin size of 500 Kb. Visual analysis of *Ampli*1^™^ LowPass profiles confirmed the high concordance to aCGH profiles (Fig 5a and 5b; S20–S25 Figs). Moreover, *Ampli*1^™^ LowPass showed high accuracy in calling of aCGH-detected CNAs with AUCs ranging between 0.81 and 0.91 for cell lines NCI-H1650, NCI-H23, NCI-H2228 and NCI-H1563 (Fig 5c and 5l). A lower agreement between *Ampli*1^™^ LowPass and aCGH was observed for lines NCI-H441 and hyperhexaploid NCI-H661. However, visual analysis of the former line shows noisy and flatter profiles for aCGH, which may contribute for problems in CNA callings (S24 Fig); the latter mainly shows differences in segmentation and CNA calling despite the similar profiles and a pretty good correlation of copy number profiles (0.84≤R^2^≤0.87; S25 and S26 Figs). This is likely due to a compression of copy number alterations due the multiploid nature of cell line NCI-H661, which might hinders an accurate calling of CNAs.

Finally, low-pass shows a high concordance with aCGH also from a quantitative point of view as indicated by the high correlation between copy number values of low-pass CNA calls compared with corresponding aCGH fold changes (R^2^ = 0.89; S27 Fig).

### Determination of single cell ploidy

Different bioinformatic methods are already available to determine cancer ploidy and purity such as ABSOLUTE [50], ASCAT [51], THetA [52] and PyLOH [53]. The first two are however designed for SNP array data and do not formally model DNA sequencing data, THetA is designed to identify subclonal CNAs in mixed samples data obtained by high throughput sequencing (40X coverage) and is thus not suitable to low-pass sequencing data, PyLOH uses allelic information which is not available for low-pass sequencing data.

**Fig 6:**
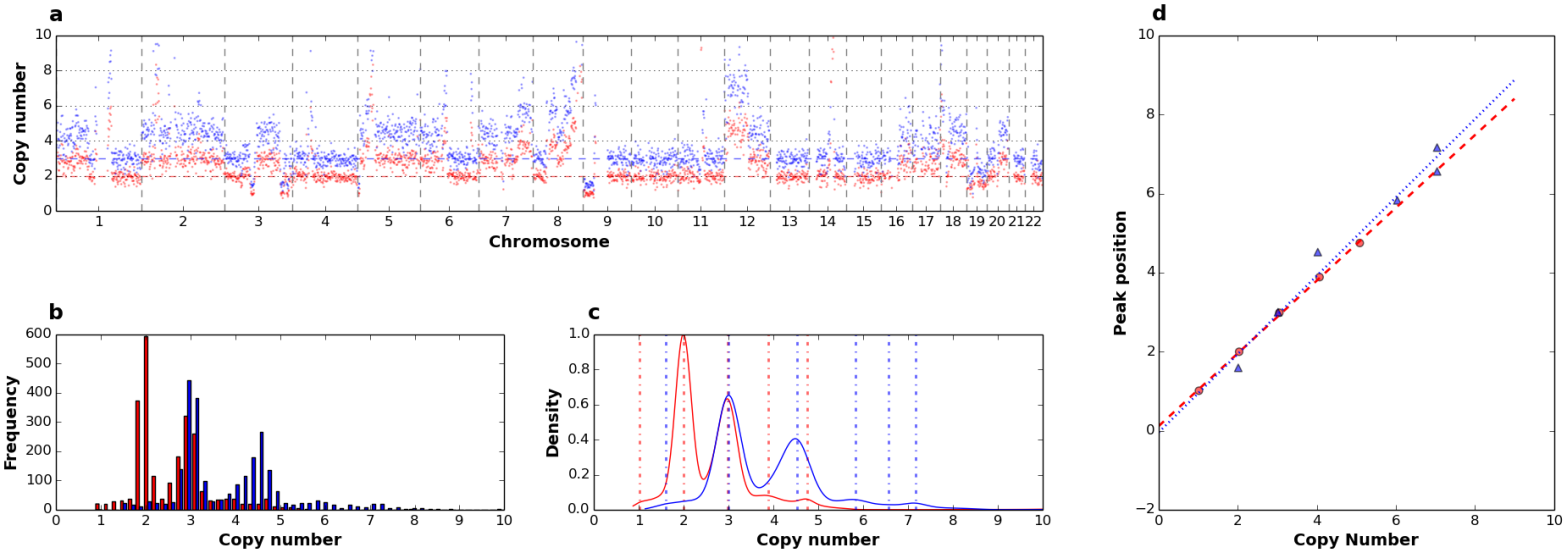
Determination of single cell ploidy. Analysis of one single cell from the near-diploid cell line NCI-H23 analyzed using a main ploidy of 2 (red) and 3 (blue): a) copy number profiles along 22 chromosomes; b) copy number levels distribution; c) density estimated by KDE; peaks detected are indicated as dashed vertical lines; d) linear regression of peak values over putative underlying copy numbers: clearly peaks obtained with a main ploidy of 2 better approximate the regression line compared to those obtained at a main ploidy of 3.

**Fig 7:**
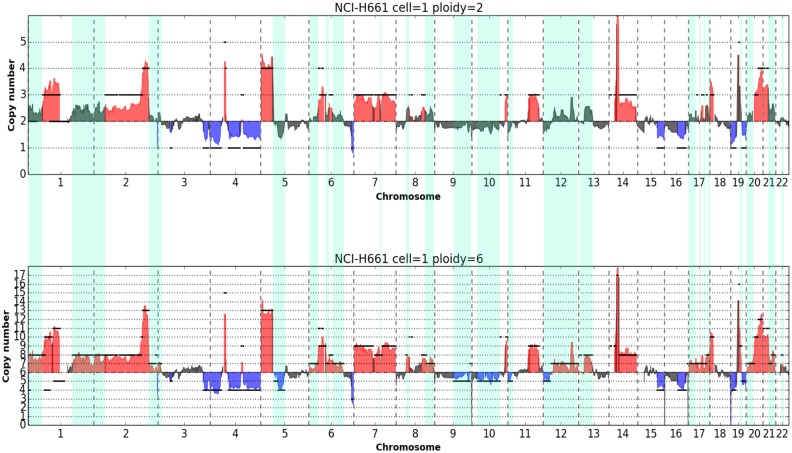
Absolute copy number CNA calling in a single cell of hyperhexaploid cell line NCI-H661. Plots of copy number profiles along the 22 autosomes expressed as absolute copy numbers. In a) and b) profiles obtained from the same sequencing data with main ploidy parameter set to 2 and 6 respectively. Significant copy number gains and losses are highlighted in red and blue respectively. Clearly a main cell ploidy = 6 provides a better fit of profiles with segmented data (black lines) and improves CNA calling. CNA calls only detected with main ploidy = 6 are shaded in green.

The method we present exploits the linear relationship between read counts and underlying copy numbers [39]. Indeed, it is expected that, for a given cell ploidy, normalized ratios of gains and losses will scale linearly with read counts. We illustrate this in Fig 6 where copy number profiles were generated from the analysis of a single cell from aberrant cell line NCI-H23 by using 2 different main ploidies (Fig 6a). Frequency distribution of smoothed copy number data, obtained by multiplying normalized ratio by the main ploidy, show a multimodal distribution where different modes ideally correspond to different copy number levels in the genome of the cell analyzed and highest peak correspond to the main ploidy (Fig 6b). Kernel density estimation and determination of modes by a peak detection method based on wavelet transform [41] clearly shows that a main ploidy of 2 produces a better fit to hypothetic underlying copy numbers, compared to 3. Indeed peaks for a main ploidy of 2 are = (1.0, 1.97, 2.94, 3.82, 4.67) and are reasonably centered around the putative underlying ploidies (1, 2, 3, 4, 5). On the contrary peaks detected with a main ploidy = 3 are = (1.58, 2.98, 4.49, 5.79, 6.52, 7.12). As expected, regression analysis of peak positions vs putative underlying copy numbers shows a ploidy of 2 produces a better fit with higher R^2^ (1.0) and SSR (0.008) compared to a main ploidy of 3 (R^2^ = 0.97; SSR = 0.668). We tested the method on *Ampli*1^™^ LowPass data from 2 single cell of the hyperesaploid cell line NCI-H661 (S28 Fig) using 7 different main ploidies (2, 3, 4, 5, 6, 7, 8). Best fits were obtained with main ploidies set to 6, in agreement to what suggested from cytogenetic data available for the cell line. Absolute copy number plots obtained from one single cell from cell line NCI-H661 by setting alternatively a main ploidy of 2 and 6 clearly show that segments better represent the underlying copy number profiles with a main ploidy of 6 (Fig 7, S29 Fig). Moreover, setting a ploidy of 6 improves CNA calling by doubling (108% increase) regions called as gains or losses which were missed because of compression effects as can be clearly seen from visual analysis of profiles.

### Copy-number profiling of CTCs

To test the assay on real clinical samples, we analyzed single CTCs and corresponding control white blood cells (WBC) from 1 patient with prostate cancer and 2 patients with lung cancer for a total of 15 CTCs and 7 WBCs. Cells were sorted with DEPArray^™^ system and genomic DNA was processed with *Ampli*1^™^ LowPass workflow. In average 854,484 reads were sequenced for each sample and data were analyzed according to the bioinformatics protocol outlined (S1 Table).

**Fig 8:**
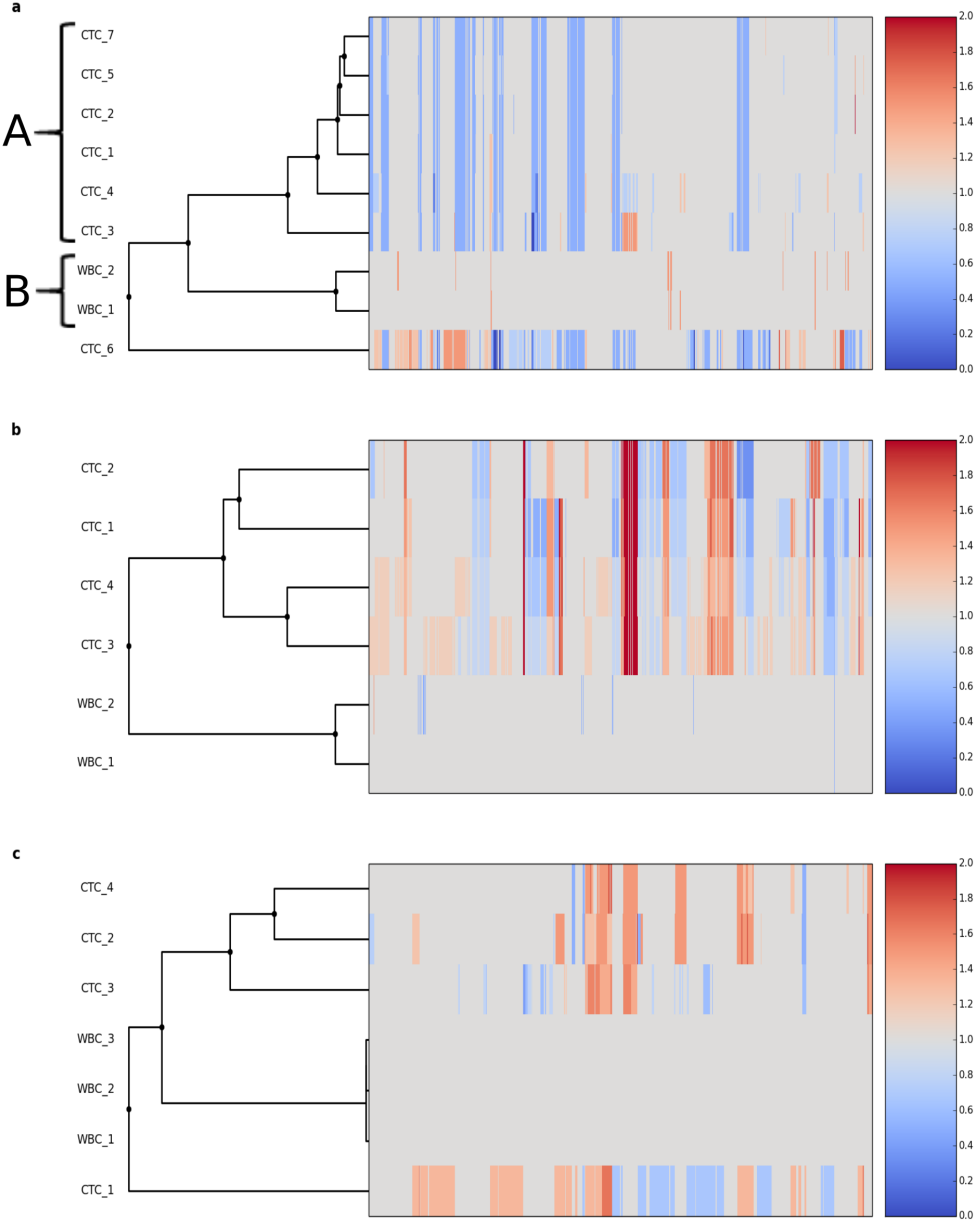
Cluster analysis of copy number profiles for CTCs and WBCs from 3 patients. a) single cells (CTCs and WBCs) from a patient affected by prostate cancer; cluster A represents 6 CTCs with small or no differences in copy number profiles; cluster B is formed by WBCs clustering, as expected, on a distinct branch of the tree. b,c) single cells (CTCs and WBCs) from 2 patients affected by lung adenocarcinoma. Values are expressed as fold changes respect to the main ploidy.

Prostate cancer CTCs and WBCs were collected from a patient with late stage metastatic castration- resistant disease with increasing CTC counts despite therapy prostate cancer. For all the CTCs a main ploidy of 2 was predicted and CNA analysis showed, on average, 660 Mbp of the genome impacted by copy gains and losses (S30 Fig). Conversely, WBCs showed a flat profile. Notably, total amount of copy number alterations ranged from 536 Mbp to over 1.2 Gbp. Cluster analysis of copy number profiles highlighted a main group of 6 CTCs with small or no differences among different cells (Fig 8a; cluster A), a cluster (B) corresponding to the 2 WBCs and a single CTC (CTC 6) showing a divergent profile compared to the main group of CTCs. Systematic analysis of CNAs revealed 15 losses common to all the cells of cluster. Most of these ‘core’ alterations were also present in CTC 6 (74% of length) indicating a common aberrant genetic background for all the cells analyzed. At the same time the analysis shows also alterations specific to single CTCs: single cell CTC 3, while belonging to the cluster of the 6 most similar CTCs and sharing all the losses with them, have a large copy gain accounting for more than 120 Mbp on chromosome 8 and CTC 6 has 534 Mbp of copy gains not present on the other cells (Fig 8a).

The second patient studied suffered from lung adenocarcinoma. All the 4 CTCs were assigned ploidies > 2 with alterations covering from 1.18 Gbp to 1.64 Gbp of genome (S31 Fig, Fig 8b). Profiles clearly showed a common genomic background with 42% of losses with respect to the main ploidy and 23% of gains with respect to the main ploidy shared among all the cells.

The third dataset was also obtained from a patient affected by lung adenocarcinoma; in this case all the 4 CTCs were assigned a ploidy > 2. Despite the limited number of cells analyzed the method highlights a huge cell-to-cell variation (S32 Fig; Fig 8c). Globally 2.06 Gbp of genome have a variation respect to the main ploidy in at least 1 of the 4 CTCs. However only 127 Mbp ‘core’ gains are shared among all the CTCs and CTC-specific CNAs range in size from 617 Mbp to 1668 Mbp. Again this demonstrates that *Ampli1*^™^ LowPass is able to capture the huge heterogeneity and cell-to-cell variation between different CTCs of the same patient.

## Discussion

Application of single-cell molecular profiling to tumor diagnostics and genome-informed therapeutics, requires high-throughput, highly-reproducible, straightforward methods. Our approach offers a streamlined, robust method for copy number profiling of single cancer cells. By exploiting the universal sequences present at *Ampli*1^™^ WGA products end, a simple PCR reaction, with appropriate hybrid primers encompassing the WGA-primer at the 3’ end, enables the introduction of barcoded NGS-adaptors, conveniently substituting several enzymatic reactions present in standard library preparation protocols, such as WGA adaptors removal, fragmentation, A-tailing and ligation [31,32]. This allowed us to reduce the efforts needed to more rapidly produce sequencing-ready libraries substantially decreasing workflow time to allow higher throughput, while reducing costs from expensive enzymatic reactions.

Another important parameter affecting the efficacy/cost ratio of NGS-based assays is the number of reads employed. Higher number of reads theoretically can produce higher resolutions and accuracy of copy number profiles, while sensibly affecting the cost of the assay. By performing a saturation analysis on data obtained from single cells from an aberrant cell line, we found that 200,000 reads are sufficient to detect CNAs with an accuracy comparable to 3.5 million of reads at a resolution of 100 Kbp. Thus, our approach allowed us to decrease cost of analysis from about $300/sample for aCGH (using widely available SurePrint G3 Human CGH Microarray, 4x180K) to about $30, including the generation of about 200.000 sequencing reads per sample on the Ion S5 platform and Ion 530 Chip, or to about $50, including sequencing of 500.000 reads, while providing performances comparable or superior to aCGH. Indeed, comparison with aCGH showed high concordance between *Ampli*1^™^ LowPass profiles and aCGH, which is a widely utilized platform for CNA analysis in tumor biopsies, with advantages regarding the ability of *Ampli*1^™^ LowPass to determine absolute copy numbers [54]. This has important implications for the biological interpretation of cancer samples, where it is important not only to determine relative copy number changes from the main ploidy but it is even more important to determine absolute copy numbers [50]. Finally, as sequencing cost per base will likely decrease in the future with advancements in sequencing technologies, the cost of copy number analysis will further diminish in the future.

Our approach, which is based on LM-PCR WGA, is superior, in principle, to other NGS-based solutions for high-throughput copy number profiling, such as the one recently proposed by Baslan *et al*., based on DOP-PCR [33]. Indeed, previous reports have shown that LM-PCR WGA approach achieves an accurate and more even representation of the original single-cell genomic DNA compared to available methods [27,43,44] and that it enables the detection of CNAs from single cells by aCGH with lower derivative log ratio spread (DLRS) value and a better call rates [55,56]. Moreover, the method has been shown to be superior to DOP-PCR for the analysis of copy-number profiles from minute amounts of microdissected FFPE material, when using aCGH, mCGH, as well as for other genetic analysis assays such as Loss Of Heterozygosity [44]. In our study we demonstrated that *Ampli*1^™^ LowPass approach provides an unbiased and uniform view of the copy number status in whole genome. While one possible issue of ligation mediated amplification method is the non-uniform distribution of *MseI* recognition sites (*TTAA*) along the genome, we demonstrated that the highly deterministic nature of the method allows to accurately predict and correct read count biases by employing standard GC-based normalization method, already implemented in available CNA detection software. This allows *Ampli*1^™^ LowPass to seamlessly integrate in standard bioinformatics workflows and pipelines. In addition, the comparison of profiles and CNA calls from *Ampli*1^™^ LowPass analysis of single cell with those obtained by WGS of bulk gDNA from aberrant cell lines conclusively proved that the method provides an accurate representation of copy number profiles in single cells without any distortion introduced by WGA.

To test the workflow in a real world case, we applied *Ampli*1^™^ LowPass analysis to 3 sets of CTCs and WBCs from 3 patients suffering from advanced prostate or lung adenocarcinoma. Data showed a low level of noise, measured as DLRS at a resolution of 500 Kbp, with values close to those obtained from single cells from cell lines. Different CTCs from the same patient showed highly consistent results indicating a high reproducibility of the method. Partially divergent profiles were also present which may be ascribed to tumor heterogeneity and possibly represent different subpopulations that, subjected to selection, may acquire resistance to drugs. Several cancer related genes were found in “core” alterations shared across different CTCs of the same patient. Interestingly, in the prostate patient, whose core alterations included mostly subchromosomal losses, a pattern reminiscent of Homology Recombination Deficiency (HRD), a copy-number loss was detected in BRCA2 locus (on Chr 13) which, on a diploid genome, implies loss of heterozygosity. In turn, BRCA2 Loss of Heterozygosity (LOH) has been linked to sensitivity to PARP inhibitors and platinum based chemotherapy [57], thus suggesting a potential link to therapy selection. In the second patient studied, suffering from lung adenocarcinoma, all the cells had a large amplification (100 Mbp) on chromosome 8 (up to over 10 copies) harboring, among others, the c-MYC gene. c-MYC is an important member of the MYC proto-oncogene family containing N-MYC, c-MYC, and L-MYC [58]. Gene amplification or copy number gain of c-MYC have been documented in several solid tumors from different tissues [59–63]. In lung cancer, some early studies revealed frequent c-MYC amplification in small cell lung cancer cell lines [64,65]. Notably, c-MYC gain is a poor-prognostic factor for disease-free survival (DFS) and overall survival (OS) in lung adenocarcinoma [58]. Interestingly, while prostate CTCs had a base ploidy = 2, all the CTCs from patients suffering from lung adenocarcinoma were detected as polyploid. This is in agreement with previous observations as cells with supernumerary centrosomes have been observed in many tumor types, including breast cancer [66], pancreatic cancer [67], prostate cancer [68], and lung and colon carcinoma [69]. Notably, for lung adenocarcinoma, 36% of tumors have been reported to have more than 68 chromosomes [70].

In conclusion, the *Ampli*1^™^ LowPass workflow presented allows accurate copy number profiling of genome and CNA detection with a low sequencing depth. The low number of reads required makes the method ideal for multiplexed sequencing on high throughput sequencers, thus leading to a cost effective solution which, while being cheaper than aCGH, provides at least a similar level of accuracy in CNA calling. Moreover, contrary to aCGH, where the limited dynamic range and linearity do not allow direct gene copy number estimates, low-pass sequencing combined to *Ampli*1^™^ technology allows the direct estimation of ploidy of single cells and absolute copy number, greatly improving CNA detection. We envision that our approach will not only be useful for studying cancer heterogeneity and tumor evolution but, given the association of copy number levels and aneuploidy status with tumor biology, it will be a powerful tool to enable the personalized therapeutics of cancer. Indeed, recent work by Carter et al. highlighted the importance of profiling single CTCs and has shown that the molecular analysis of CTCs identifies distinct copy number profiles in patients with chemosensitive and chemorefractory small-cell lung cancer, demonstrating the clinical utility of molecular profiling of single CTCs to accurately delineate responders from non-nonresponders [32]. Moreover, independent work has shown that high amounts of somatic CNAs correlate with a reduction of immune-mediated cytotoxic and pro-inflammatory activities in the tumor micro-environment (TME), while low somatic CNA levels correlated with long term survival [71], and markers identified by genome-wide analysis of CNAs have been shown to correlate with response and resistance to immunotherapies [72]. Genome wide analysis of CNAs has, thus, the promise to discriminate responders from non-responders to allow the employment of more effective therapies.

## Supporting information

S1 Table Sequencing statistics of samples processed with *Ampli*1^™^ LowPass protocol. (XLSX)

S1 Fig Density of fragment length distribution in SC-LP-WGS data. (PDF)

S2 Fig Normalized fragment count between all WBC. Normalized fragment counts show a homogeneous and comparable distribution among WBCs. The boxes extend from the first to third quartile values of the data, with a line at the median. The upper whiskers extend to last datum lower than third quartile + 1.5 * interquartile range (IQR). The lower whiskers extend to the first datum greater than first quartile– 1.5 * IQR. Outlier points are those past the end of the whiskers.
(PDF)

S3 Fig Mappability of genomic bins. Boxplot showing mappability values of bins deviating less or more than 3 standard deviations and outlier bins. The boxes extend from the first to third quartile values of the data, with a line at the median. The upper whiskers extend to last datum lower than third quartile + 1.5 * interquartile range (IQR). The lower whiskers extend to the first datum greater than first quartile– 1.5 * IQR. Outlier points are those past the end of the whiskers.
(PDF)

S4 Fig Uniformity. Each WBC shows an uniformity value greater than 99%; 90% of bins is on average covered by >60% of normalized read counts average.
(PDF)

S5 Fig Detection of problematic regions. Median fold change across 21 control WBCs is displayed. Bins with fold change > 1.4 or < 0.6 (triangle-down) are mostly located in pericentromeric regions (shaded in grey) or near telomers.
(PDF)

S6 Fig Copy number profiles in line NCI-H1650. On top: copy number profiles obtained by WGS of bulk genomic DNA (gDNA); in the 2 plots on bottom: copy number profiles by low-pass WGS on DNA from 2 single cells amplified with *Ampli*1^™^ WGA kit. Copy number values are expressed as logged fold change. Statistically significant copy number gains are highlighted in red while statistically significant copy number losses are highlighted in blue.
(PDF)

S7 Fig Copy number profiles in line NCI-H1563. On top: copy number profiles obtained by WGS of bulk genomic DNA (gDNA); in the 2 plots on bottom: copy number profiles by low-pass WGS on DNA from 2 single cells amplified with *Ampli*1^™^ WGA kit. Copy number values are expressed as logged fold change. Statistically significant copy number gains are highlighted in red while statistically significant copy number losses are highlighted in blue.
(PDF)

S8 Fig Copy number profiles in line NCI-H23. On top: copy number profiles obtained by WGS of bulk genomic DNA (gDNA); in the 2 plots on bottom: copy number profiles by low-pass WGS on DNA from 2 single cells amplified with *Ampli*1^™^ WGA kit. Copy number values are expressed as logged fold change. Statistically significant copy number gains are highlighted in red while statistically significant copy number losses are highlighted in blue.
(PDF)

S9 Fig Copy number profiles in line NCI-H441. On top: copy number profiles obtained by WGS of bulk genomic DNA (gDNA); in the 2 plots on bottom: copy number profiles by low-pass WGS on DNA from 2 single cells amplified with *Ampli*1^™^ WGA kit. Copy number values are expressed as logged fold change. Statistically significant copy number gains are highlighted in red while statistically significant copy number losses are highlighted in blue.
(PDF)

S10 Fig Specificity at increasing reads number and windows size. Random subsamples of a pool of reads from 21 «normal» control WBCs from 7 individuals were analyzed for CNVs at different resolutions (bin size). Region in the map corresponding to a bin size of 200 Kbp and 400,000 reads is highlighted by a red box.
(PDF)

S11 Fig Copy number profiles in cell line NCI-1650 cell #1 at 1 Mbp resolution at different downsampling factors. (PDF)

S12 Fig Copy number profiles in cell line NCI-1650 cell #2 at 1 Mbp resolution at different downsampling factors. (PDF)

S13 Fig Copy number profiles in cell line NCI-1650 cell #1 at 500 Kbp resolution at different downsampling factors. (PDF)

S14 Fig Copy number profiles in cell line NCI-1650 cell #2 at 500 kbp resolution at different downsampling factors. (PDF)

S15 Fig Copy number profiles in cell line NCI-1650 cell #1 at 200 kbp resolution at different downsampling factors. (PDF)

S16 Fig Copy number profiles in cell line NCI-1650 cell #2 at 200 kbp resolution at different downsampling factors. (PDF)

S17 Fig Copy number profiles in cell line NCI-1650 cell #1 at 100 kbp resolution at different downsampling factors. (PDF)

S18 Fig Copy number profiles in cell line NCI-1650 cell #2 at 100 kbp resolution at different downsampling factors. (PDF)

S19 Fig Regression analysis for LPCNA experiments at different read depths and resolution. (PDF)

S20 Fig Comparison of copy number profiles in NCI-H1650 single cells generated by low-pass sequencing and aCGH. Low-pass sequencing and aCGH were performed starting from DNA from 2 single cells processed with *Ampli*1^™^ WGA kit. Copy number gains and losses are highlighted in red and blue respectively.
(PDF)

S21 Fig Comparison of copy number profiles in NCI-H23 single cells generated by low-pass sequencing and aCGH. Low-pass sequencing and aCGH were performed starting from DNA from 2 single cells processed with *Ampli*1^™^ WGA kit. Copy number gains and losses are highlighted in red and blue respectively.
(PDF)

S22 Fig Comparison of copy number profiles generated in NCI-H2228 single cells by low-pass sequencing and aCGH. Low-pass sequencing and aCGH were performed starting from DNA from 2 single cells processed with *Ampli*1^™^ WGA kit. Copy number gains and losses are highlighted in red and blue respectively.
(PDF)

S23 Fig Comparison of copy number profiles in NCI-H1563 single cells generated by low-pass sequencing and aCGH. Low-pass sequencing and aCGH were performed starting from DNA from 2 single cells processed with *Ampli*1^™^ WGA kit. Copy number gains and losses are highlighted in red and blue respectively.
(PDF)

S24 Fig Comparison of copy number profiles in NCI-H441 single cells generated by low-pass sequencing and aCGH. Low-pass sequencing and aCGH were performed starting from DNA from 2 single cells processed with *Ampli*1^™^ WGA kit. Copy number gains and losses are highlighted in red and blue respectively.
(PDF)

S25 Fig Comparison of copy number profiles in NCI-H661 single cells generated by low-pass sequencing and aCGH. Low-pass sequencing and aCGH were performed starting from DNA from 2 single cells processed with *Ampli*1^™^ WGA kit. Copy number gains and losses are highlighted in red and blue respectively.
(PDF)

S26 Fig Correlation between aCGH and LowPass logFC values in cell line NCI-H661. For both single cells NCI-H661-1 and NCI-H661-2 the LowPass copy number data (expressed as logged fold change on base 2) show an high correlation with aCGH.
(PDF)

S27 Fig Correlation of LP vs aCGH logFC for common CNAs. Only CNAs of length ≥ 500Kb were considered.
(PDF)

S28 Fig Determination of single cell ploidy in a hyperhesaploid cell line. Analysis of one single cell from the hyperesaploid cell line NCI-H661 analyzed using a main ploidy of 2 (red) and 6 (blue): a) copy number profiles along 22 chromosomes; b) copy number levels distribution; c) results of density estimation by KDE; peaks detected are indicated with a dashed vertical line; d) linear regression of peak values over putative underlying copy numbers.
(PDF)

S29 Fig Absolute copy number CNA calling in a single cell of hyperhexaploid cell line NCI-H661 (cell #2). Plots of copy number profiles along the 22 autosomes expressed as absolute copy numbers. In a) and b) profiles obtained from the same sequencing data with main ploidy parameter set to 2 and 6 respectively. Significant copy number gains and losses are highlighted in red and blue respectively. Clearly a main cell ploidy = 6 provides a better fit of profiles with segmented data (black lines) and improves CNA calling. CNA calls only detected with main ploidy = 6 are shaded in green.
(PDF)

S30 Fig Copy number profiles in CTCs and WBCs of a patient affected by prostate cancer. On X axis is the position on the 22 autosomes, while on Y axis is the absolute copy number. Each dot represents a window (500 Kbp). Significant gains are highlighted in red, while losses are highlighted in blue.
(PDF)

S31 Fig Copy number profiles from CTCs and WBCs of a patient affected by lung adenocarcinoma. On X axis is the position on the 22 autosomes, while on Y axis is the absolute copy number. Each dot represents a window (500 Kbp). Significant gains are highlighted in red, while losses are highlighted in blue.
(PDF)

S32 Fig Copy number profiles from CTCs and WBCs of a patient affected by lung adenocarcinoma. On X axis is the position on the 22 autosomes, while on Y axis is the absolute copy number. Each dot represents a window (500 Kbp). Significant gains are highlighted in red, while losses are highlighted in blue.
(PDF)
