# Supplementary figures and images for: A streamlined workflow for single-cells genome-wide copy-number profiling by low-pass sequencing of LM-PCR whole-genome amplification products
Source: PLoS One. 2018 Mar 1;13(3):e0193689. doi: 10.1371/journal.pone.0193689 (PMC5832318; doi:10.1371/journal.pone.0193689)

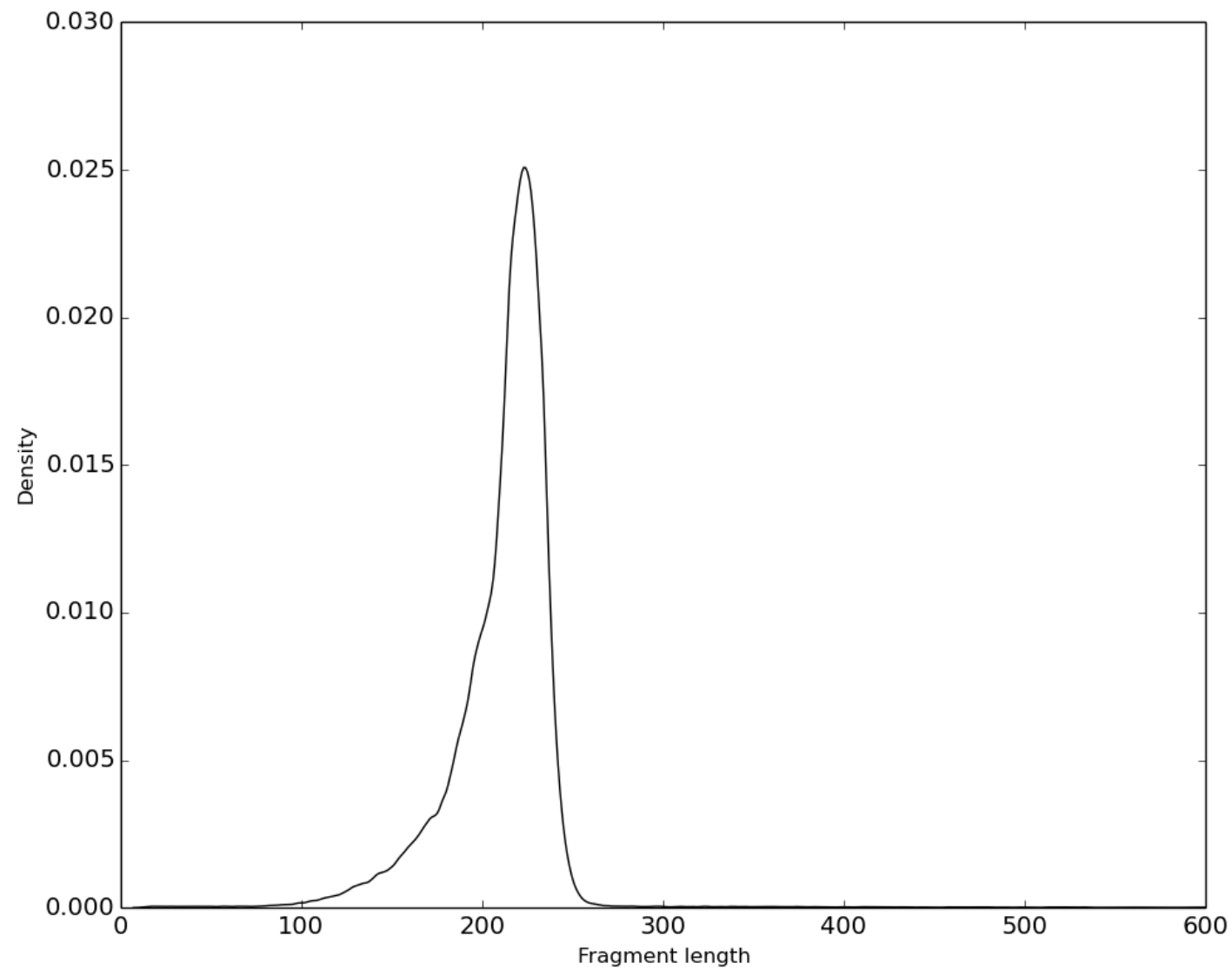

**S1 Figure: Density of fragment length distribution in LP-WGS data of a single WBC.**

Supplement: S1 Fig — (PDF) [file pone.0193689.s002.pdf]

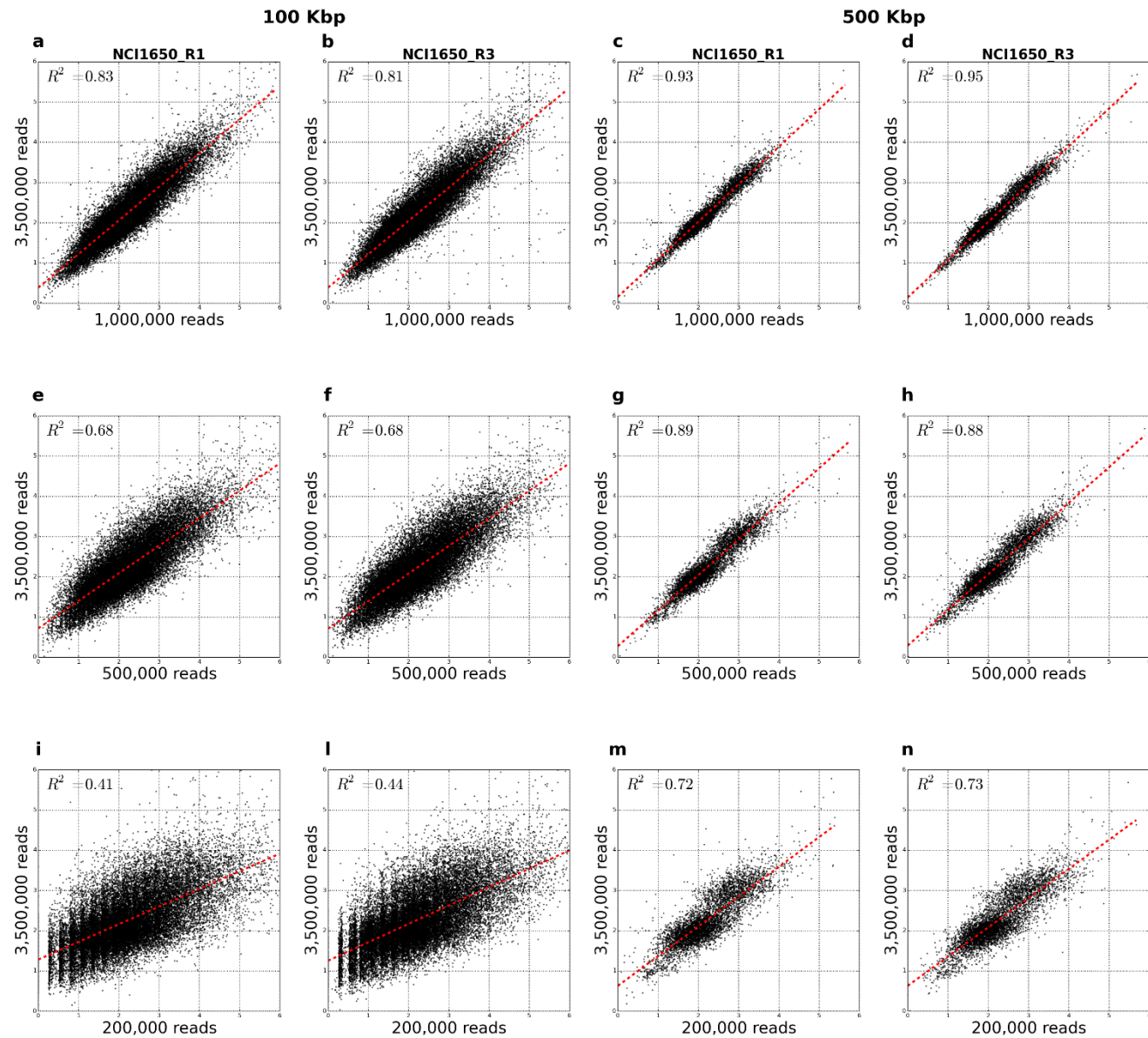

**S19 Figure: regression analysis for LPCNA experiments at different read depths and resolution.**

Supplement: S19 Fig — (PDF) [file pone.0193689.s020.pdf]
