# Supplementary material for: A streamlined workflow for single-cells genome-wide copy-number profiling by low-pass sequencing of LM-PCR whole-genome amplification products
Source: PLoS One. 2018 Mar 1;13(3):e0193689. doi: 10.1371/journal.pone.0193689 (PMC5832318; doi:10.1371/journal.pone.0193689)
Supplement: S2 Fig — Normalized fragment counts show a homogeneous and comparable distribution among WBCs. The boxes extend from the first to third quartile values of the data, with a line at the median. The upper whiskers extend to last datum lower than third quartile + 1.5 * interquartile range (IQR). The lower whiskers extend to the first datum greater than first quartile– 1.5 * IQR. Outlier points are those past the end of the whiskers. (PDF) [file pone.0193689.s003.pdf]

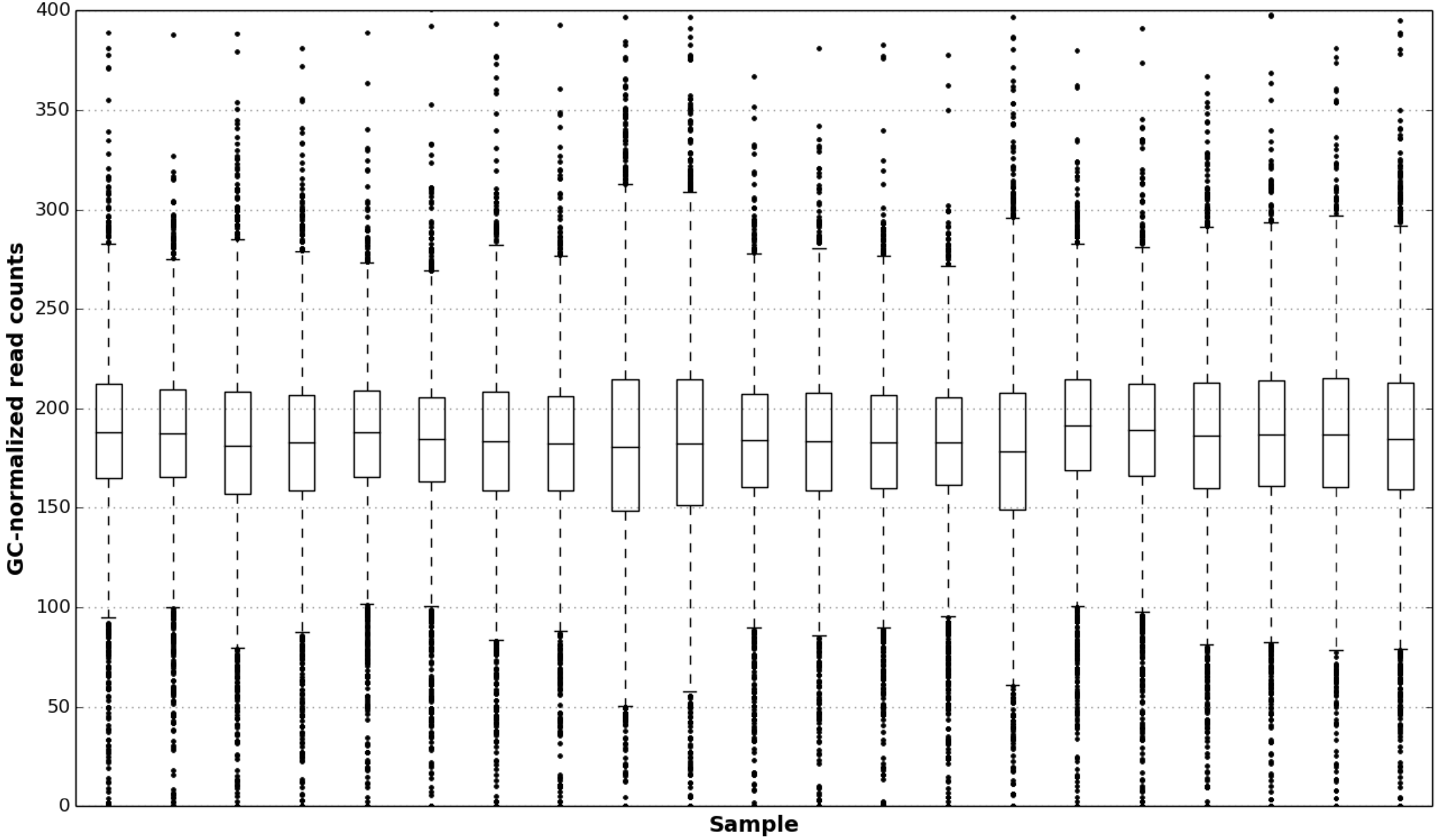

S2 Figure: Normalized read counts between all WBC. Normalized read counts show a homogeneous and comparable distribution among WBCs. The boxes extend from the first to third quartile values of the data, with a line at the median. The upper whiskers extend to last datum lower than third quartile + 1.5 * interquartile range (IQR). The lower whiskers extend to the first datum greater than first quartile – 1.5 * IQR. Outlier points are those past the end of the whiskers.
