# Supplementary material for: A streamlined workflow for single-cells genome-wide copy-number profiling by low-pass sequencing of LM-PCR whole-genome amplification products
Source: PLoS One. 2018 Mar 1;13(3):e0193689. doi: 10.1371/journal.pone.0193689 (PMC5832318; doi:10.1371/journal.pone.0193689)
Supplement: S3 Fig — Boxplot showing mappability values of bins deviating less or more than 3 standard deviations and outlier bins. The boxes extend from the first to third quartile values of the data, with a line at the median. The upper whiskers extend to last datum lower than third quartile + 1.5 * interquartile range (IQR). The lower whiskers extend to the first datum greater than first quartile– 1.5 * IQR. Outlier points are those past the end of the whiskers. (PDF) [file pone.0193689.s004.pdf]

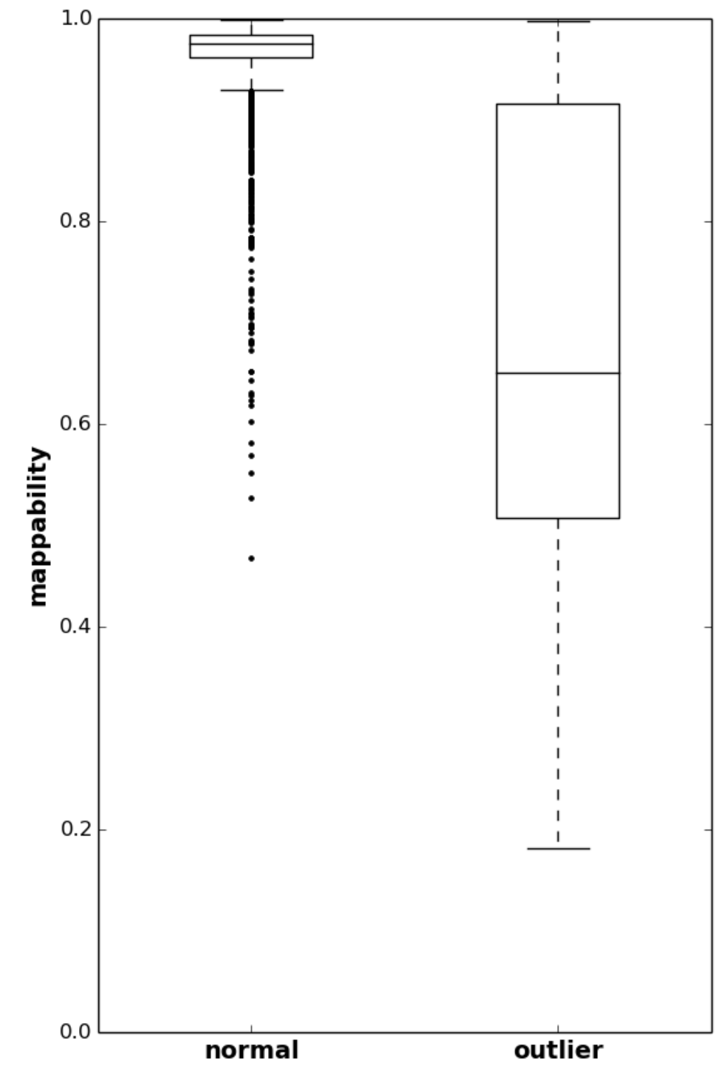

S3 Figure: Mappability of genomic bins. Boxplot showing mappability values of bins deviating less (normal) or more (outlier) than 3 standard deviations. The boxes extend from the first to third quartile values of the data, with a line at the median. The upper whiskers extend to last datum lower than third quartile + 1.5 * interquartile range (IQR). The lower whiskers extend to the first datum greater than first quartile – 1.5 * IQR. Outlier points are those past the end of the whiskers.
