# Supplementary material for: A streamlined workflow for single-cells genome-wide copy-number profiling by low-pass sequencing of LM-PCR whole-genome amplification products
Source: PLoS One. 2018 Mar 1;13(3):e0193689. doi: 10.1371/journal.pone.0193689 (PMC5832318; doi:10.1371/journal.pone.0193689)
Supplement: S4 Fig — Each WBC shows an uniformity value greater than 99%; 90% of bins is on average covered by >60% of normalized read counts average. (PDF) [file pone.0193689.s005.pdf]

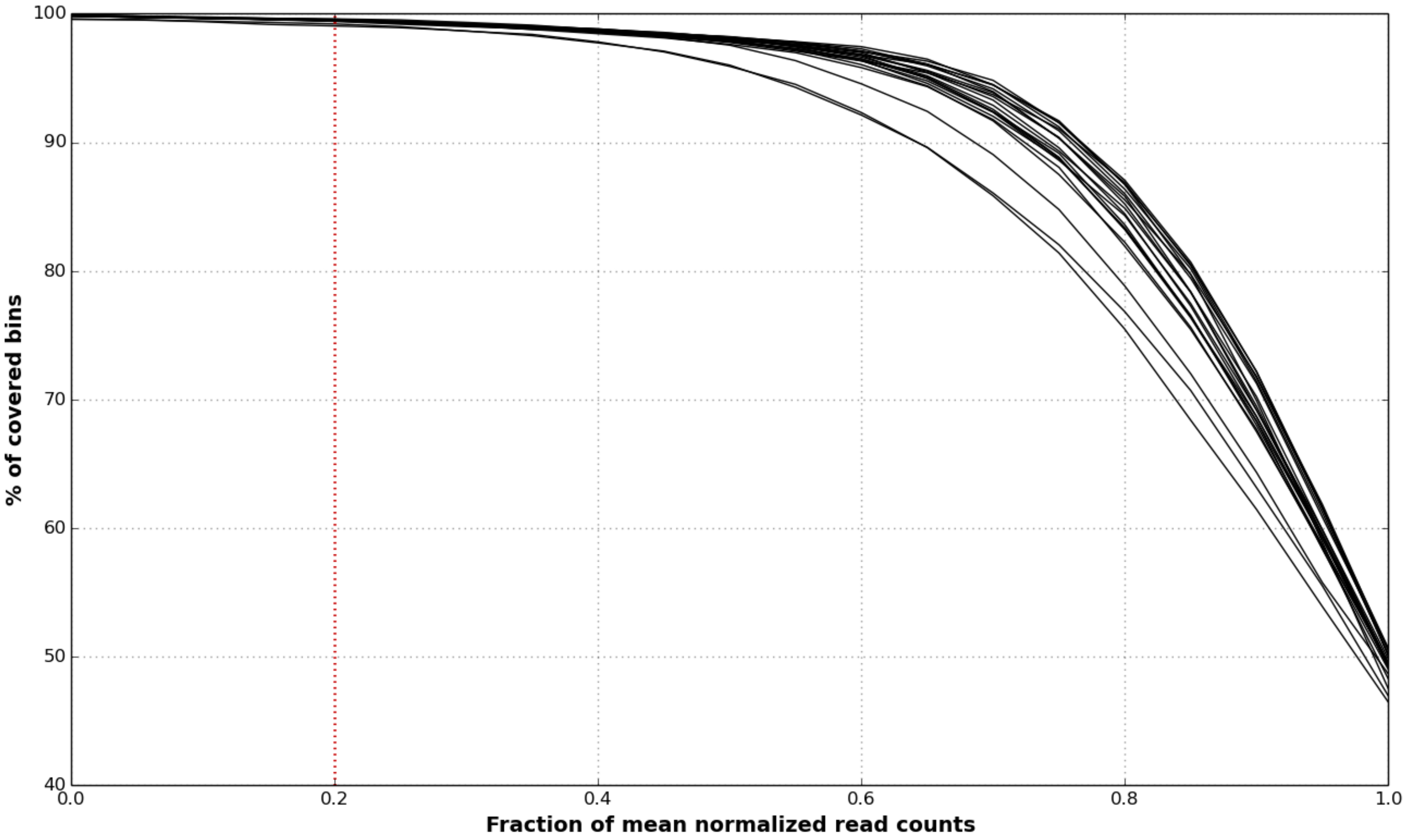

S4 Figure: Uniformity. Each WBC shows an uniformity value greater than 99%; 90% of bins is on average covered by >60% of normalized read counts average.
