# Supplementary material for: A streamlined workflow for single-cells genome-wide copy-number profiling by low-pass sequencing of LM-PCR whole-genome amplification products
Source: PLoS One. 2018 Mar 1;13(3):e0193689. doi: 10.1371/journal.pone.0193689 (PMC5832318; doi:10.1371/journal.pone.0193689)
Supplement: S5 Fig — Median fold change across 21 control WBCs is displayed. Bins with fold change > 1.4 or < 0.6 (triangle-down) are mostly located in pericentromeric regions (shaded in grey) or near telomers. (PDF) [file pone.0193689.s006.pdf]

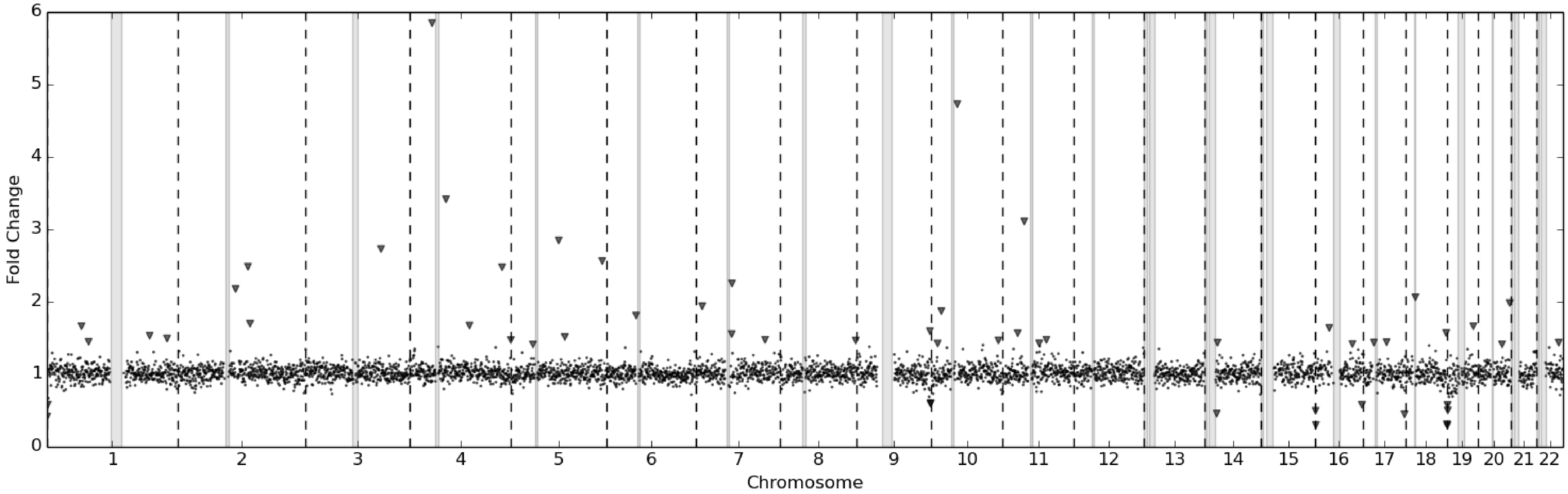

S5 Figure: Detection of problematic regions. Median fold change across 21 control WBCs is displayed. Bins with fold change > 1.4 or < 0.6 (triangle-down) are mostly located in pericentromeric regions (shaded in grey) or near telomers.
