# Supplementary material for: A streamlined workflow for single-cells genome-wide copy-number profiling by low-pass sequencing of LM-PCR whole-genome amplification products
Source: PLoS One. 2018 Mar 1;13(3):e0193689. doi: 10.1371/journal.pone.0193689 (PMC5832318; doi:10.1371/journal.pone.0193689)
Supplement: S9 Fig — On top: copy number profiles obtained by WGS of bulk genomic DNA (gDNA); in the 2 plots on bottom: copy number profiles by low-pass WGS on DNA from 2 single cells amplified with Ampli1™ WGA kit. Copy number values are expressed as logged fold change. Statistically significant copy number gains are highlighted in red while statistically significant copy number losses are highlighted in blue. (PDF) [file pone.0193689.s010.pdf]

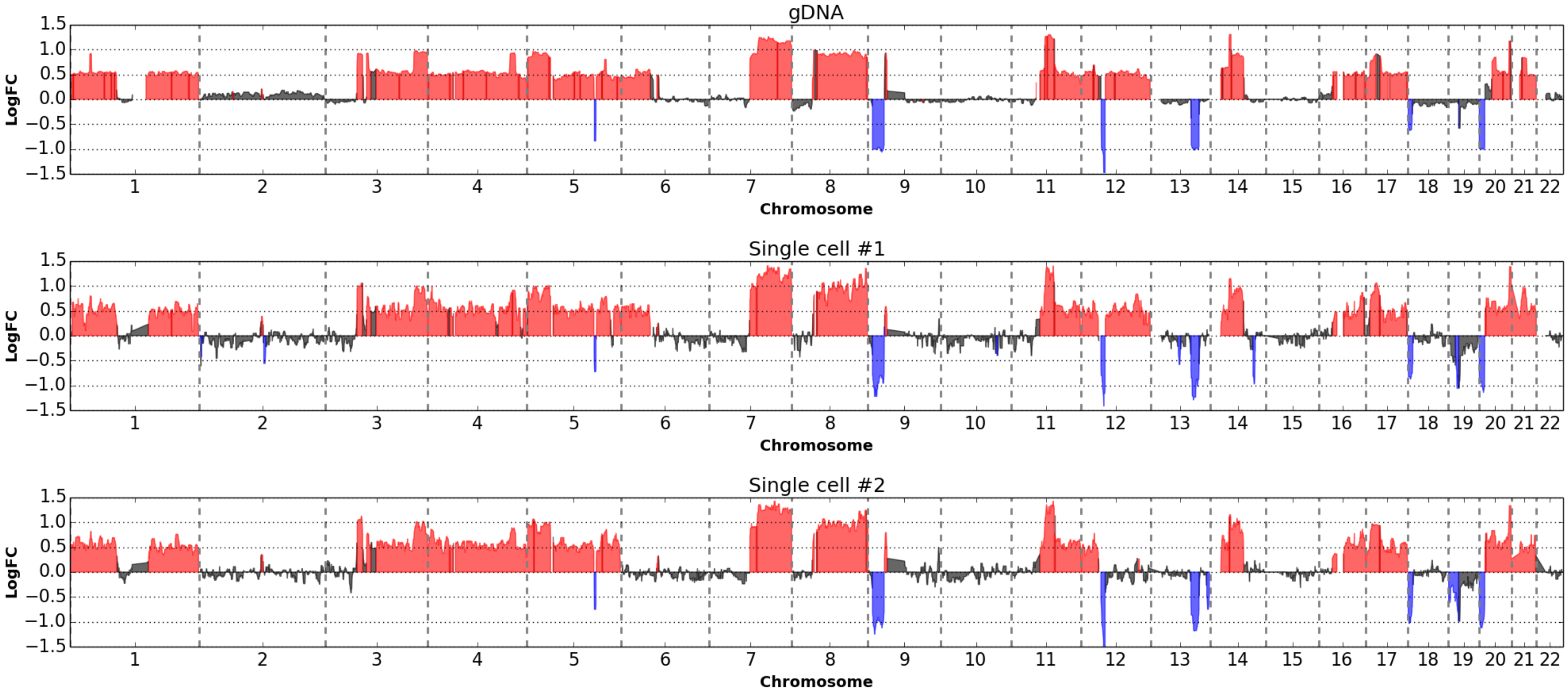

S9 Figure: Copy number profiles in line NCI-H441. On top: copy number profiles obtained by WGS of bulk genomic DNA (gDNA); in the 2 plots on bottom: copy number profiles by low-pass WGS on DNA from 2 single cells amplified with *Ampli*1™ WGA kit. Copy number values are expressed as logged fold change. Statistically significant copy number gains are highlighted in red while statistically significant copy number losses are highlighted in blue.
