# Supplementary material for: A streamlined workflow for single-cells genome-wide copy-number profiling by low-pass sequencing of LM-PCR whole-genome amplification products
Source: PLoS One. 2018 Mar 1;13(3):e0193689. doi: 10.1371/journal.pone.0193689 (PMC5832318; doi:10.1371/journal.pone.0193689)
Supplement: S10 Fig — Random subsamples of a pool of reads from 21 «normal» control WBCs from 7 individuals were analyzed for CNVs at different resolutions (bin size). Region in the map corresponding to a bin size of 200 Kbp and 400,000 reads is highlighted by a red box. (PDF) [file pone.0193689.s011.pdf]

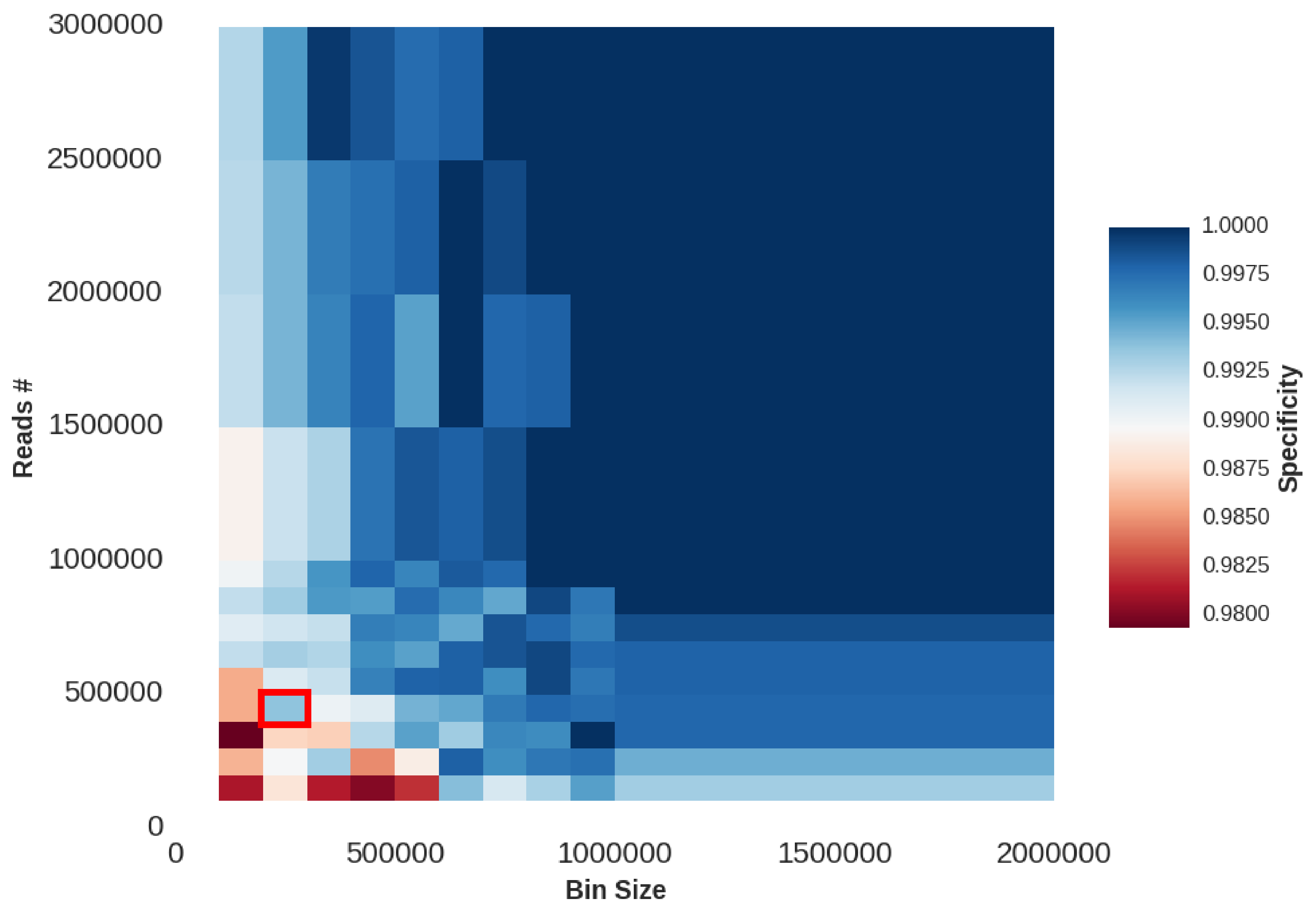

S10 Figure: Specificity at increasing reads number and windows size. Random subsamples of a pool of reads from 21 «normal» control WBCs from 8 individuals were analyzed for CNAs at different resolutions (bin size). Region in the map corresponding to a bin size of 200 Kbp and 400,000 reads is highlighted by a red box.
