# Supplementary material for: A streamlined workflow for single-cells genome-wide copy-number profiling by low-pass sequencing of LM-PCR whole-genome amplification products
Source: PLoS One. 2018 Mar 1;13(3):e0193689. doi: 10.1371/journal.pone.0193689 (PMC5832318; doi:10.1371/journal.pone.0193689)
Supplement: S17 Fig — (PDF) [file pone.0193689.s018.pdf]

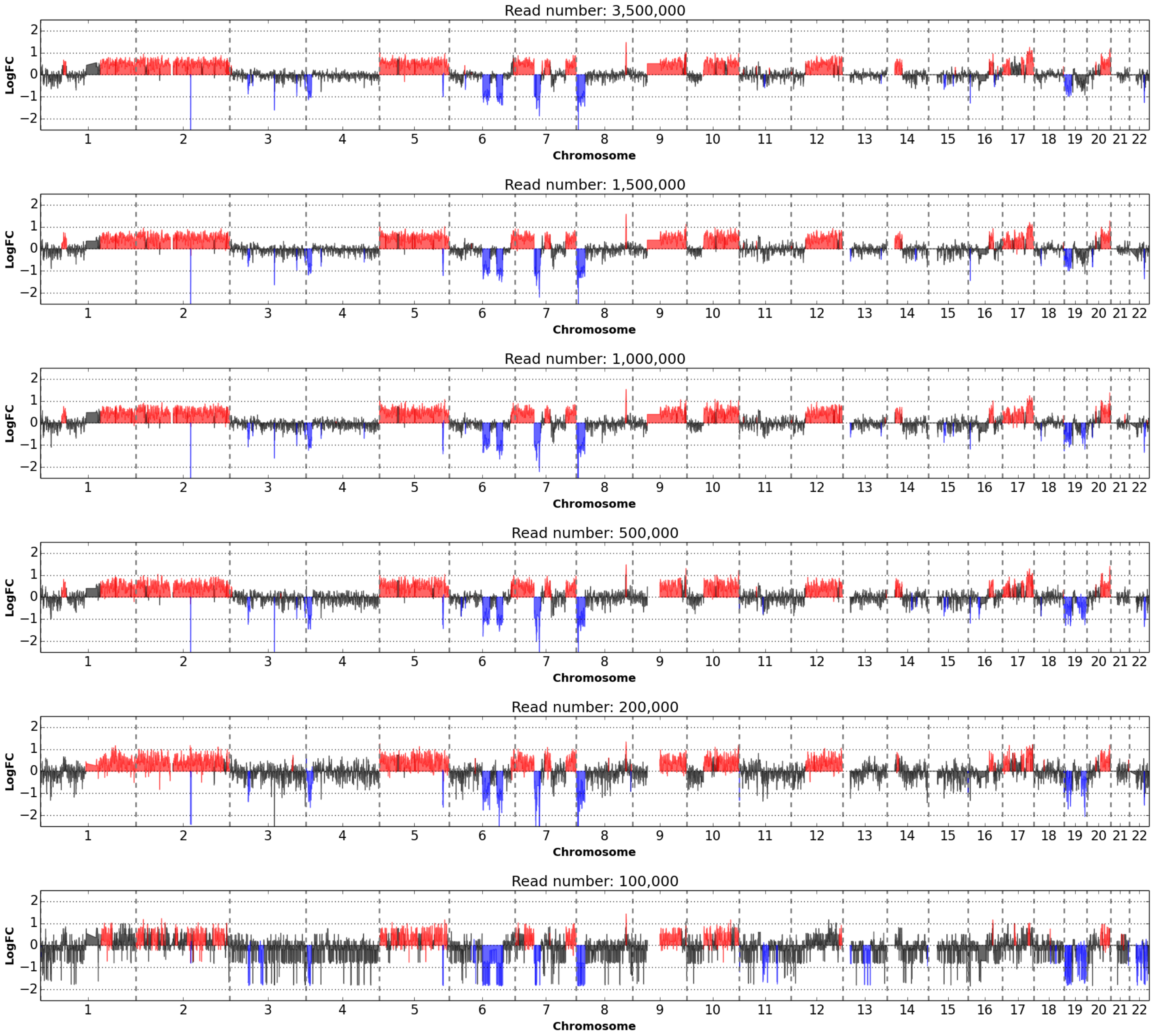

S17 Figure: Copy number profiles in cell line NCI-1650 cell #1 at 100 kbp resolution at different downsampling factors.
