# Supplementary material for: A streamlined workflow for single-cells genome-wide copy-number profiling by low-pass sequencing of LM-PCR whole-genome amplification products
Source: PLoS One. 2018 Mar 1;13(3):e0193689. doi: 10.1371/journal.pone.0193689 (PMC5832318; doi:10.1371/journal.pone.0193689)
Supplement: S22 Fig — Low-pass sequencing and aCGH were performed starting from DNA from 2 single cells processed with Ampli1™ WGA kit. Copy number gains and losses are highlighted in red and blue respectively. (PDF) [file pone.0193689.s023.pdf]

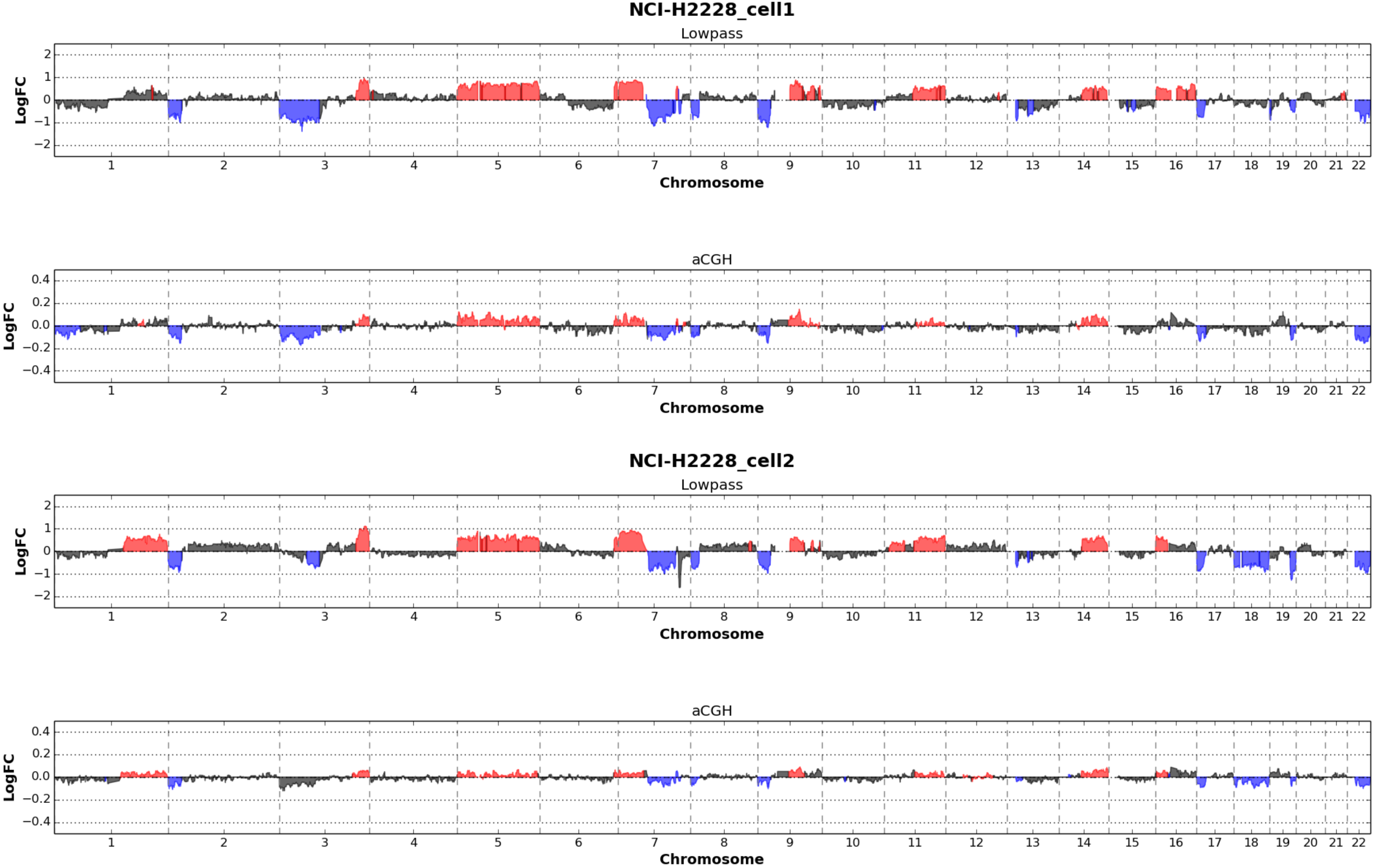

S22 Figure: Comparison of copy number profiles generated in NCI-H2228 single cells by low-pass sequencing and aCGH. Low-pass sequencing and aCGH were performed starting from DNA from 2 single cells processed with *Ampli*1™ WGA kit. Copy number gains and losses are highlighted in red and blue respectively.
