# Supplementary material for: A streamlined workflow for single-cells genome-wide copy-number profiling by low-pass sequencing of LM-PCR whole-genome amplification products
Source: PLoS One. 2018 Mar 1;13(3):e0193689. doi: 10.1371/journal.pone.0193689 (PMC5832318; doi:10.1371/journal.pone.0193689)
Supplement: S26 Fig — For both single cells NCI-H661-1 and NCI-H661-2 the LowPass copy number data (expressed as logged fold change on base 2) show an high correlation with aCGH. (PDF) [file pone.0193689.s027.pdf]

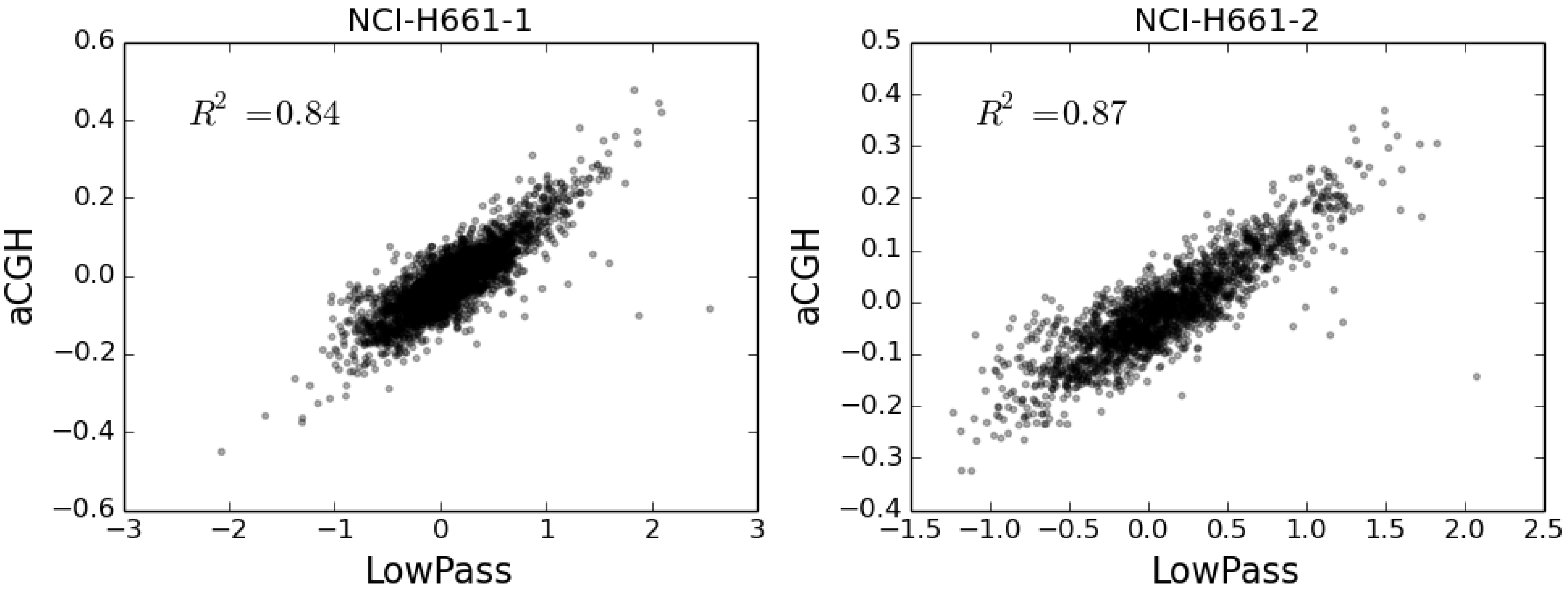

S26 Figure: Correlation between aCGH and LowPass logFC values in cell line NCI-H661. For both single cells NCI-H661-1 and NCI-H661-2 the LowPass copy number data (expressed as logged fold change on base 2) show an high correlation with aCGH.
