# Supplementary material for: A streamlined workflow for single-cells genome-wide copy-number profiling by low-pass sequencing of LM-PCR whole-genome amplification products
Source: PLoS One. 2018 Mar 1;13(3):e0193689. doi: 10.1371/journal.pone.0193689 (PMC5832318; doi:10.1371/journal.pone.0193689)
Supplement: S28 Fig — Analysis of one single cell from the hyperesaploid cell line NCI-H661 analyzed using a main ploidy of 2 (red) and 6 (blue): a) copy number profiles along 22 chromosomes; b) copy number levels distribution; c) results of density estimation by KDE; peaks detected are indicated with a dashed vertical line; d) linear regression of peak values over putative underlying copy numbers. (PDF) [file pone.0193689.s029.pdf]

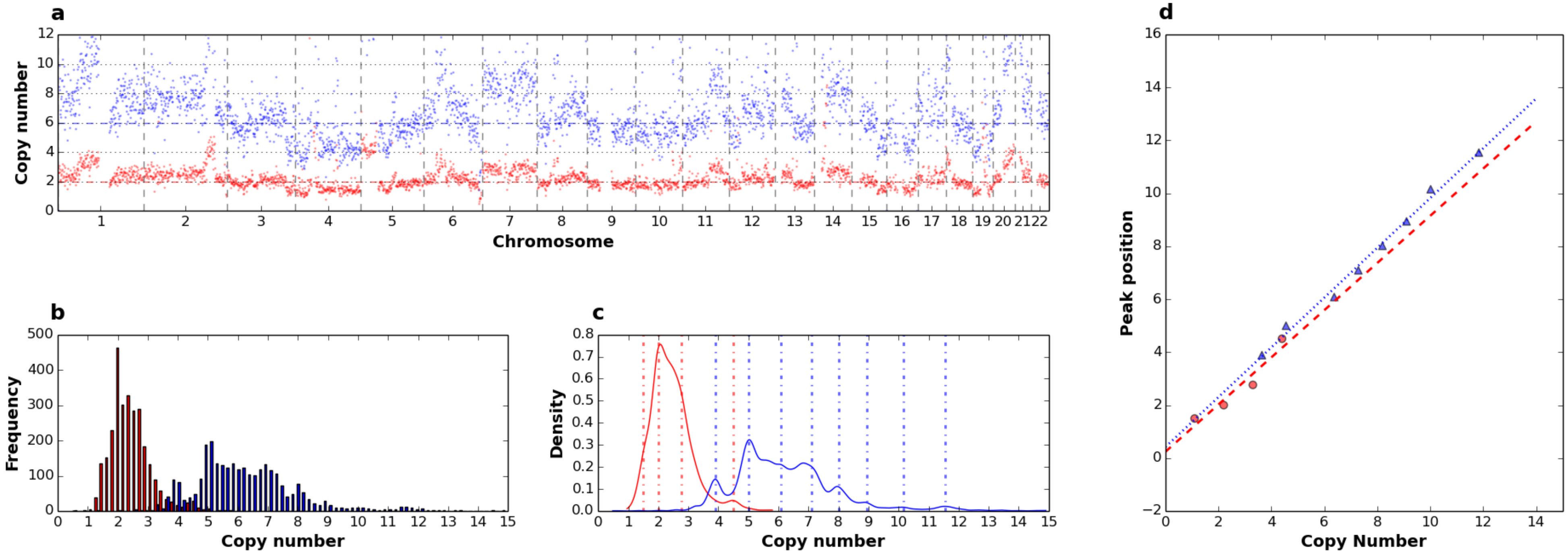

S28 Figure: Determination of single cell ploidy in a hyperhesaploid cell line. Analysis of one single cell from the hyperesaploid cell line NCI-H661 analyzed using a main ploidy of 2 (red) and 6 (blue): a) copy number profiles along 22 chromosomes; b) copy number levels distribution; c) results of density estimation by KDE; peaks detected are indicated with a dashed vertical line; d) linear regression of peak values over putative underlying copy numbers.
