# Supplementary material for: A streamlined workflow for single-cells genome-wide copy-number profiling by low-pass sequencing of LM-PCR whole-genome amplification products
Source: PLoS One. 2018 Mar 1;13(3):e0193689. doi: 10.1371/journal.pone.0193689 (PMC5832318; doi:10.1371/journal.pone.0193689)
Supplement: S29 Fig — Plots of copy number profiles along the 22 autosomes expressed as absolute copy numbers. In a) and b) profiles obtained from the same sequencing data with main ploidy parameter set to 2 and 6 respectively. Significant copy number gains and losses are highlighted in red and blue respectively. Clearly a main cell ploidy = 6 provides a better fit of profiles with segmented data (black lines) and improves CNA calling. CNA calls only detected with main ploidy = 6 are shaded in green. (PDF) [file pone.0193689.s030.pdf]

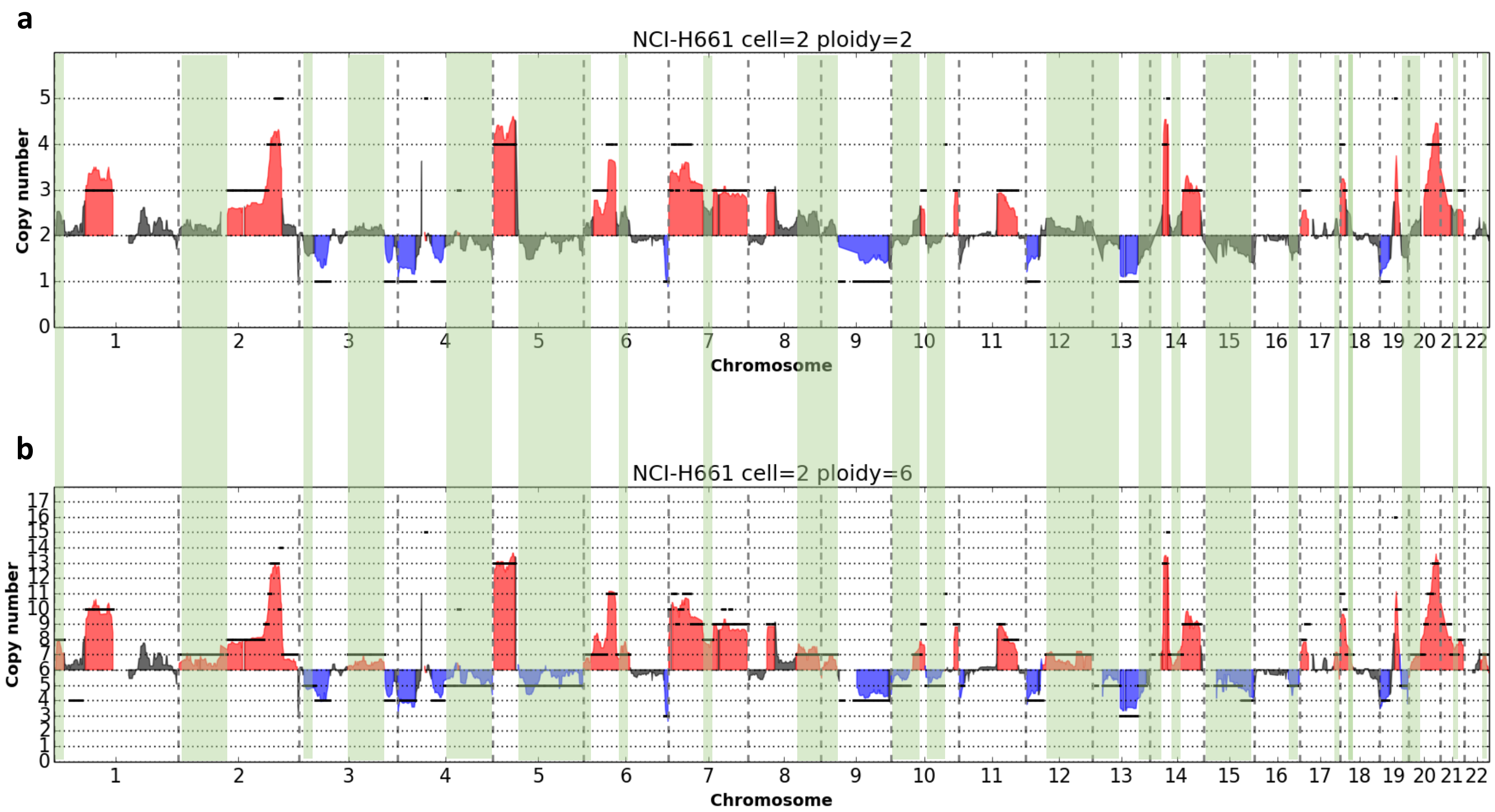

S29 Figure: Absolute copy number CNA calling in a single cell of hyperhexaploid cell line NCI-H661 (cell #2)
Plots of copy number profiles along the 22 autosomes expressed as absolute copy numbers. In a) and b) profiles obtained from the same sequencing data with main ploidy parameter set to 2 and 6 respectively. Significant copy number gains and losses are highlighted in red and blue respectively. Clearly a main cell ploidy = 6 provides a better fit of profiles with segmented data (black lines) and improves CNA calling. CNA calls only detected with main ploidy = 6 are shaded in green
