# Supplementary material for: A streamlined workflow for single-cells genome-wide copy-number profiling by low-pass sequencing of LM-PCR whole-genome amplification products
Source: PLoS One. 2018 Mar 1;13(3):e0193689. doi: 10.1371/journal.pone.0193689 (PMC5832318; doi:10.1371/journal.pone.0193689)
Supplement: S31 Fig — On X axis is the position on the 22 autosomes, while on Y axis is the absolute copy number. Each dot represents a window (500 Kbp). Significant gains are highlighted in red, while losses are highlighted in blue. (PDF) [file pone.0193689.s032.pdf]

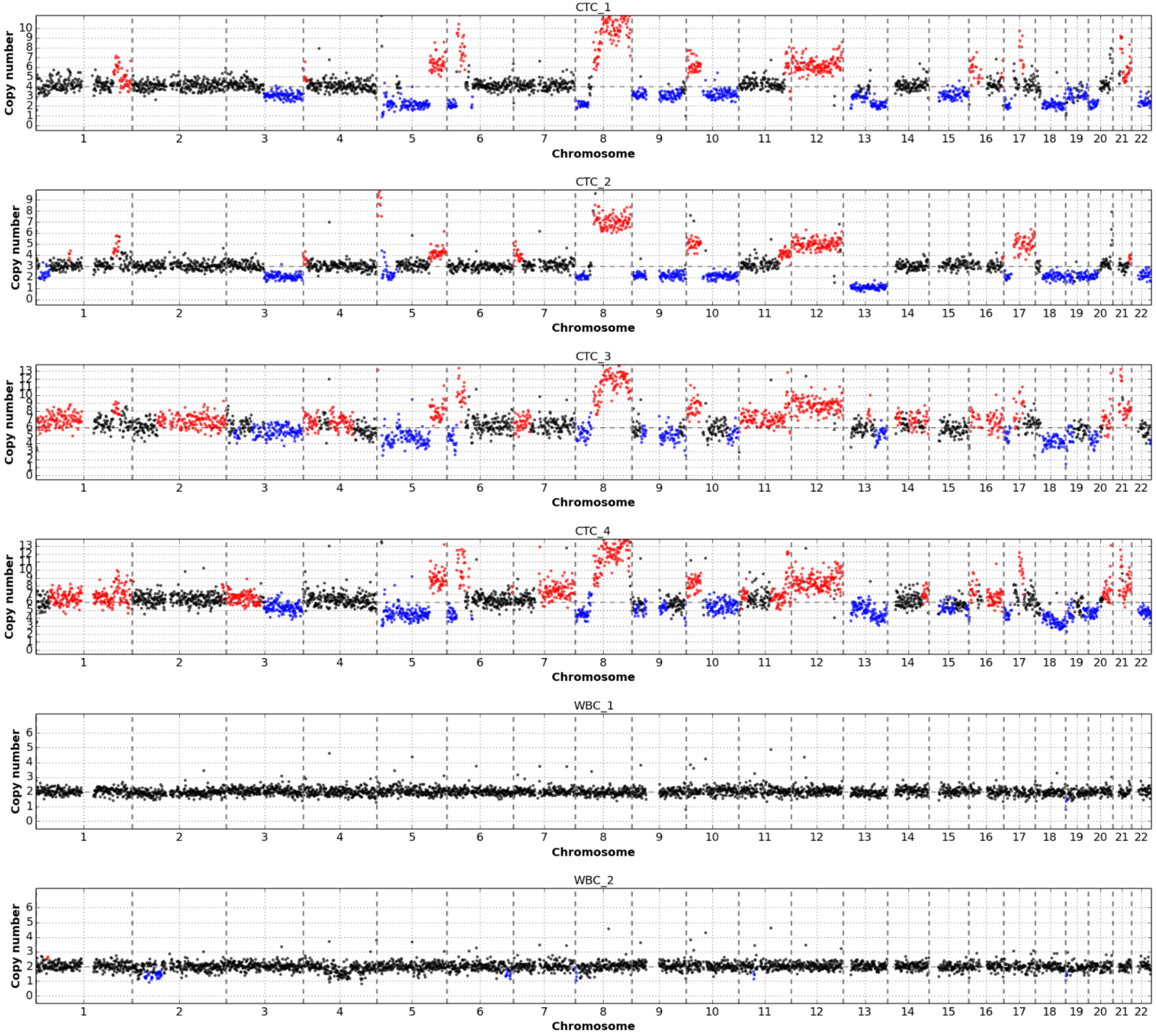

S31 Figure: Copy number profiles from CTCs and WBCs of a patient affected by lung adenocarcinoma. On X axis is the position on the 22 autosomes, while on Y axis is the absolute copy number. Each dot represents a window (500 Kbp). Significant gains are highlighted in red, while losses are highlighted in blue.
